# Spike sorting with Kilosort4

**DOI:** 10.1038/s41592-024-02232-7

**Published:** 2024-04-08

**Authors:** Marius Pachitariu, Shashwat Sridhar, Jacob Pennington, Carsen Stringer

**Affiliations:** 1 HHMI, Ashburn, VA USA; 2 Department of Ophthalmology, University Medical Center Göttingen, Göttingen, Germany; 3 Department of Mathematics, Washington State University, Vancouver, WA USA

**Keywords:** Computational neuroscience, Computational platforms and environments

## Abstract

Spike sorting is the computational process of extracting the firing times of single neurons from recordings of local electrical fields. This is an important but hard problem in neuroscience, made complicated by the nonstationarity of the recordings and the dense overlap in electrical fields between nearby neurons. To address the spike-sorting problem, we have been openly developing the Kilosort framework. Here we describe the various algorithmic steps introduced in different versions of Kilosort. We also report the development of Kilosort4, a version with substantially improved performance due to clustering algorithms inspired by graph-based approaches. To test the performance of Kilosort, we developed a realistic simulation framework that uses densely sampled electrical fields from real experiments to generate nonstationary spike waveforms and realistic noise. We found that nearly all versions of Kilosort outperformed other algorithms on a variety of simulated conditions and that Kilosort4 performed best in all cases, correctly identifying even neurons with low amplitudes and small spatial extents in high drift conditions.

## Abstract (web summary)

Kilosort4 is a spike-sorting algorithm with improved performance compared to previous versions, owing to the use of a graph-based clustering approach. The tool extracts the activity of individual neurons from electrophysiological recordings acquired with, for example, Neuropixels electrodes.

## Main

Classical spike-sorting frameworks require a sequence of operations, which can be categorized into preprocessing, spike detection, clustering and postprocessing. Modern approaches have improved these steps by introducing new algorithms. Some frameworks^1–3^ took advantage of new clustering algorithms such as density-based approaches^4^ or agglomerative approaches using bimodality criteria^5^. In contrast, the original Kilosort^6^ used a simple clustering approach (scaled *k*-means) but combined two steps of the pipeline into one (spike detection + clustering = template learning) and added an extra matching pursuit step for detecting overlapping spikes, sometimes referred to as solving the ‘collision problem’^7–12^.

These early algorithms for large-scale electrophysiology required substantial human curation, as the clustering results were imperfect. This was mainly due to the nonstationary nature of data from real experiments. The electrical field of a unit sampled by a probe, called a spike waveform, should be fixed and reproducible across long time periods. Yet in many experiments, the shape of the waveform seemed to change over the course of hours and sometimes much faster. The main reason for these changes was identified as vertical probe movement or ‘drift’, using high-density electrodes^13^. Correcting for drift resulted in substantial improvements in spike-sorting performance (see Methods in ref. ^13^).

The main goal of this paper is to describe Kilosort4 and demonstrate its performance. Some of the algorithmic steps in Kilosort4 (see Table 1 for an overview) are directly inherited from previous versions, so we do not describe them in detail here (drift correction^13^ and matching pursuit^6^). The main algorithm introduced in Kilosort4 is a graph-based clustering approach based on modularity optimization. We combined this approach with a merging tree strategy that uses meta information (such as refractory period violations and projection bimodality) to make merge/split decisions. We describe Kilosort4 in detail and benchmark it against other algorithms.

**Table 1:** The evolution of Kilosort

		Preprocessing		Template deconvolution				Clustering and postprocessing		
Algorithms	Language	Filtering and whitening	Drift correction	Template deconvolution	Template learning	Deconvolution during learning	New templates from residual	Clustering	Splits	Merges
Kilosort1 (2016)	MATLAB +CUDA	Yes^6^	−	Yes^6^	Scaled *k*-means^6^	−	−	−	−	−
Kilosort2 (2018)	MATLAB +CUDA	Yes	− (only **tracking**^**a**^)	Yes	Scaled *k*-means	**Yes**^a^	**Threshold** **crossing**^**a**^	−	**Bimodality** **pursuit**^a^	**Yes**^**a**^
Kilosort2.5 (2020)	MATLAB/**Python** +CUDA	Yes	**Yes**^13^	Yes	Scaled *k*-means	Yes	Threshold crossing	−	Bimodality pursuit	Yes
Kilosort3 (2021)	MATLAB +CUDA	Yes	Yes	Yes	**Recursive** **pursuit**^**a**^	−	−	**Recursive** **pursuit**^**a**^	Yes	Yes
Kilosort4 (2023)	Python +**pytorch**	Yes	Yes	Yes	**Graph** **clustering**^**a**^	−	−	**Graph** **clustering**^**a**^	**Merging** **tree**^**a**^	Yes

Bold text indicates new features added after Kilosort1, in the version where they were first introduced.

^a^Described in this paper.

## Results

**Extended Data Fig. 1:**
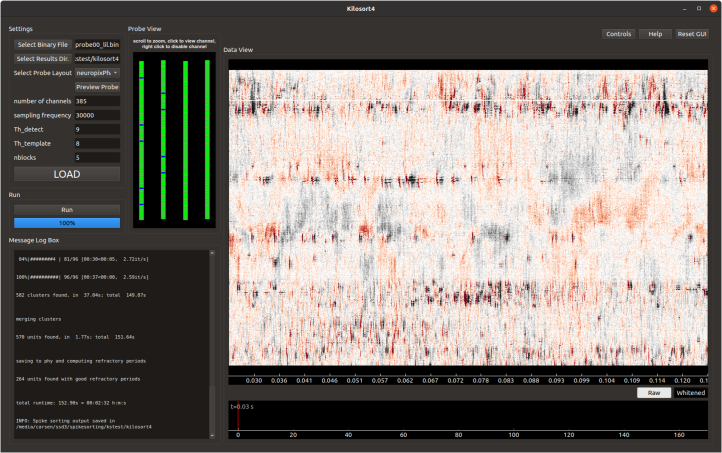
Kilosort4 graphical user interface. The GUI for Kilosort4 enables the user to load in and view the binary file both raw and whitened. Next the user runs the spike-sorting pipeline. The message log box allows the user to monitor the progress of the spike-sorting algorithm.

At the core of Kilosort4 lies a graph-based clustering algorithm, which we describe below. Before that, however, we describe the feature extraction pipeline that provides the input to the clustering algorithm. We leave the description of the graphical user interface (GUI) (Extended Data Fig. 1) and the practical implementation details to the Methods section.

### Template deconvolution

**Fig. 1:**
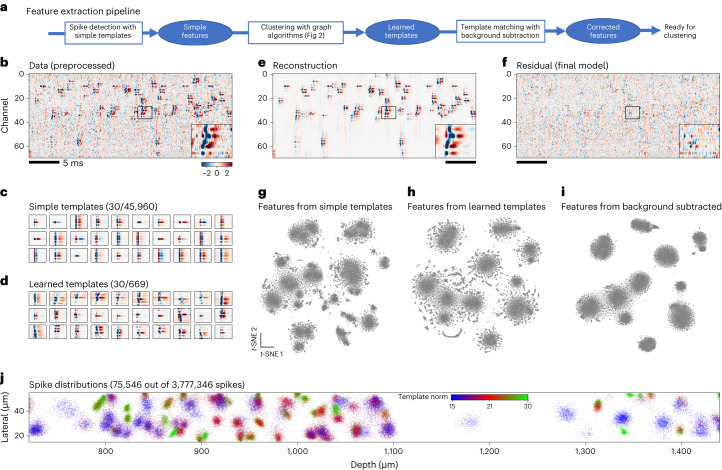
Spike detection and feature extraction. **a**, Schematic of the pipeline for detecting spikes and extracting spike features. **b**, Short segment of preprocessed data over 70 channels and 1,000 time points (data from elsewhere^16^). Insets show an expanded section with multiple overlapping spikes. **c**, Example simple templates centered at a single position on the probe. Templates are repeated at 1,536 positions for a Neuropixels probe. **d**, Example learned templates centered at different positions on the probe. **e**, Reconstruction of the data in **b** based on the inferred templates and spike times. **f**, Residual after subtracting the reconstruction from the data. **g**–**i**, *t*-SNE visualization of spike features from a 40-μm segment of the probe. Spike features were extracted using either simple templates (**g**) or learned templates without (**h**) or with (**i**) background subtraction. **j**, Spatial distribution of a subset of the final extracted spikes colored by their template norm.

The goals of the feature extraction pipeline (Fig. 1a) are to (1) detect all spikes, including overlapping ones; and (2) extract spike features after subtracting the influence of the background. We refer to the spike detection and feature extraction steps jointly as ‘template deconvolution’. This module generates a set of templates that correspond to the average spatiotemporal waveforms of neurons in the recording. The templates are used in the matching pursuit step for detecting overlapping spikes^6^. A template deconvolution step has been used in all versions of Kilosort and the background-corrected spike features have been used for visualization in Phy^14^. In Kilosort4, we go one step further and use the background-corrected features as inputs to a more powerful clustering algorithm.

**Extended Data Fig. 2:**
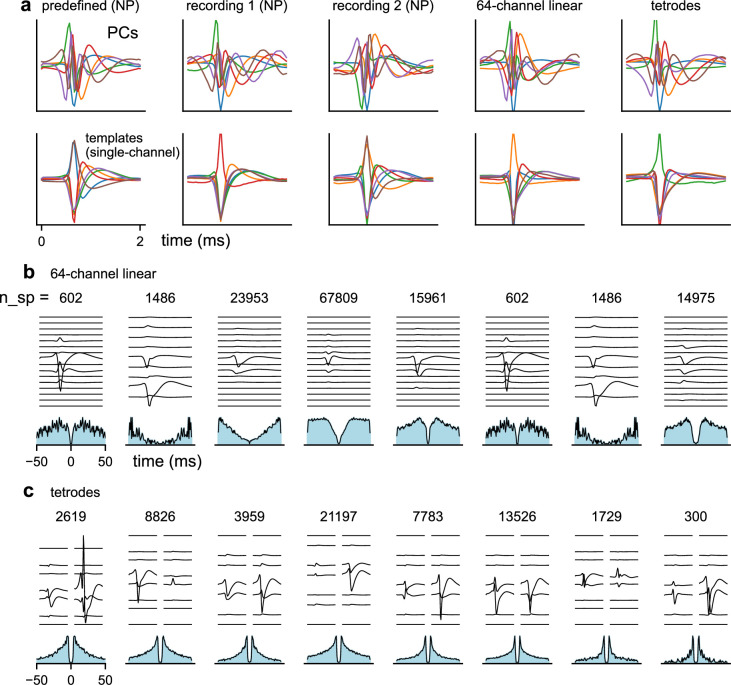
Kilosort adapts to other probes. **a**, Single-channel principal components (PCs) and templates identified from either the entire IBL repository (predefined), from two individual neuropixel recordings, from a 64-channel linear probe or from tetrodes. **b**, Eight out of 65 single units identified from a public dataset recorded with a 64-channel linear probe. (top) Waveforms across channels. Panel title represents the number of spikes for that unit. (bottom) Autocorrelograms. **c**, Same as **a** for eight single units out of 127 identified in a publicly available tetrode dataset. Groups of 4 nearby channels form a tetrode and are mostly independent from other tetrodes.

We illustrate the template deconvolution process using a recording shared by the Neuropixels paper^15,16^, containing the visual cortex, hippocampus and thalamus. First, a set of initial spike waveforms are extracted from preprocessed data using a set of simple templates that are designed to span a wide range of spatial positions, spatial sizes and waveform shapes (Fig. 1b,c and elsewhere^13^). The waveform shapes are extracted from the recording by *k*-means clustering of single-channel waveforms (Extended Data Fig. 2a). To extract features from the spikes, we use a set of principal components (PCs) identified also from the single-channel waveforms (Extended Data Fig. 2a). The spike PC features are then clustered using the graph-based algorithm from Kilosort4 (described in the next section). The centroids of the clusters are the ‘learned templates’, which are then aligned temporally (Fig. 1d). The templates are compared to each other by cross-correlation and similar templates are merged together to remove duplicates. The learned templates are then used in the matching pursuit step, which iteratively finds the best-matching templates to the preprocessed data and subtracts off their contribution. The subtraction is a critical part of the matching pursuit and allows the algorithm to detect spikes that were overlapped by the subtracted ones. Among the major current spike-sorting platforms, Kilosort is the only one that performs this subtraction, which allows it to resolve spike collisions better than all other approaches^12^. The final reconstruction of the data with the templates is shown in Fig. 1e. The residual is the difference between the data and the reconstruction and can be informative if the algorithm fails to find some units (Fig. 1f).

Unlike previous versions, Kilosort4 does not further use the templates as putative clusters; they are completely discarded after spike extraction. This is because more powerful clustering algorithms can be applied to the spike features once they have been extracted with template deconvolution. Figure 1g–i shows the *t*-distributed stochastic neighbor embedding (*t*-SNE) of three different sets of features from spikes detected over a 40-μm stretch of a Neuropixels probe. The features computed with the learned templates with background subtraction (Fig. 1i) are embedded as more uniform, Gaussian-like clusters. Without background subtraction, each cluster is surrounded by a patterned envelope of points due to the contribution of overlapping spikes and these patterns can be easily mistaken for other clusters (Fig. 1g,h). The visualization in Fig. 1i can be used to get an impression of a small section of the data without performing any clustering. To visualize the distribution of spikes over a larger portion of a probe, we plot a subset of spikes at their inferred *xy* positions (Fig. 1j; see Methods for details on how the *xy* positions are inferred). The spikes are colored according to their norms, which tend to be uniform for spikes from the same unit.

### Graph-based clustering with merging trees

The core clustering algorithm in Kilosort4 is applied twice: once in the template deconvolution pipeline to learn templates and once on the deconvolved features to assign final cluster identities. The graph-based clustering approach first constructs a graph of points connected to their nearest neighbors in Euclidean space and then constructs a cost function from the graph properties to encourage the clustering of nodes. In spike sorting, an early application of graph-based approaches was superparamagnetic clustering^17^. In more recent years, the ‘modularity’ cost function has emerged as a popular choice for graph-based algorithms, which counts the number of graph edges inside a cluster and compares them to the expected number of edges from a disorganized, unclustered null model^18^.

Well-known implementations of modularity optimization are the Leiden and Louvain algorithms^19,20^. Applied directly to spike features, these established algorithms fail in at least two ways: (1) difficulty partitioning clusters with very different number of points^21^; and (2) relatively slow processing speed for hundreds of thousands of points^19^. To remedy the first problem, we developed an algorithm that combined a graph-clustering method with a ‘merging tree’ approach. The latter allowed us to inject domain knowledge into the clustering for making split/merge decisions. To improve the processing speed, which typically grows quadratically in the number of data points, we developed a landmark-based version of graph clustering which uses nearest neighbors within a subset of all data points.

**Fig. 2:**
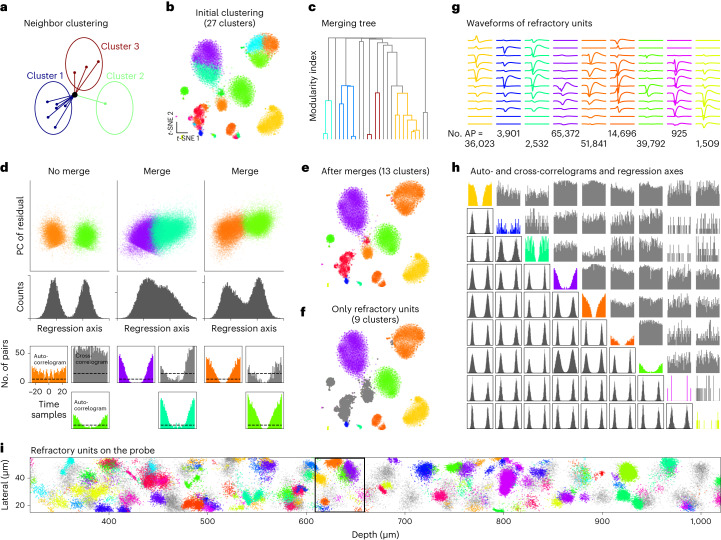
Clustering spikes with graph-based methods. **a**, Illustration of the iterative reassignment process. At each iteration, a node (black dot) is reassigned to the cluster (red, blue or green circle) that contains most of its neighbors. A penalty is used to compensate for larger clusters having more neighbors. This process is initialized with 200 clusters obtained from *k*-means++ and converges to a smaller set of clusters in tens of iterations. **b**, Example clustering produced by this process overlaid on a *t*-SNE visualization (data from elsewhere^22^). **c**, Merging tree formed by merging clusters according to the modularity cost function. Colored branches correspond to merges that were accepted. The tree is traversed from top to bottom to make split/merge decisions. **d**, Criteria for performing a merge/split decision in the merging tree: projection across the regression axis has to be bimodal (top); cross-correlogram of spike times cannot be refractory (dashed line indicates approximate refractory criterion) (bottom). **e**, Final result of the clustering algorithm after merges. **f**, Same as **e** with non-refractory units grayed out. **g**, Average waveforms of units with refractory periods and the total number of action potentials (AP) in each cluster. **h**, Autocorrelograms (diagonal); projection on regression axes (below diagonal); and cross-correlograms (above diagonal). **i**, Subset of spikes colored by their final assigned clusters. Non-refractory units are shown in gray. The black box indicates the clusters illustrated in **a**–**h**.

The graph-clustering part of the algorithm was used to obtain oversplit clusters, defined as the stationary points of an iterative neighbor reassignment algorithm based on the modularity cost function (Fig. 2a and Methods). This method allowed us to find more of the small clusters compared to a straightforward application of the Leiden algorithm (Fig. 2b). The oversplit clusters required additional merges using domain knowledge. To find the best merges, we used the modularity cost function to construct a ‘merging tree’ (Fig. 2c). The leaves of this tree correspond to the oversplit clusters and merges are sequentially identified by gradually reducing the modularity threshold. Potential splits in this tree were tested using two criteria: (1) a bimodal distribution of spike projections along the regression axis between the two subclusters (Fig. 2d, top) and (2) whether the cross-correlogram was refractory or not (Fig. 2d, bottom). These two criteria tend to be the ones most used by human curators performing spike sorting.

This clustering algorithm was applied to groups of spikes centered on a vertical segment of the probe, typically chosen as a multiple of the vertical pitch (40 μm for Neuropixels1, 30 μm for Neuropixels2, etc). Here we illustrate the process for a Neuropixels1 recording from the International Brain Laboratory (IBL) dataset^22^ containing anterior cingulate cortex, lateral septal nucleus, prelimbic cortex, striatum and corpus callosum. After all sections were clustered, an additional merging step was performed that tested the refractoriness of the cross-correlogram for all pairs of templates with a correlation above 0.5, similar to the global merging step from previous versions (2, 2.5 and 3). The final results are shown in Fig. 2e. Units that did not have a refractory period are shown grayed out in Fig. 2f; they likely correspond to neurons that were not well isolated. A quick overview of the units identified on this section of the probe shows that all units had neuronal-like waveforms and refractory autocorrelograms, all pairs of clusters had bimodal projections on their respective regression axes and all pairs of clusters had flat, non-refractory cross-correlograms (Fig. 2g,h). These properties together indicate that these nine units correspond to nine distinct, well-isolated neurons. These clusters can also be visualized on the probe in their local contexts (Fig. 2i).

### Hybrid and full simulations without drift

To test the performance of Kilosort4 and other algorithms^1–3,23,24^, we next developed a set of simulations. All algorithms other than Kilosort4 were run through their respective SpikeInterface wrappers to ensure consistent processing, and parameter adjustments were made in some cases to improve results (Methods)^25^. The latest algorithm versions as of December 2022 were used in all cases, which are often substantially different from the initial published versions^2,3^.

**Fig. 3:**
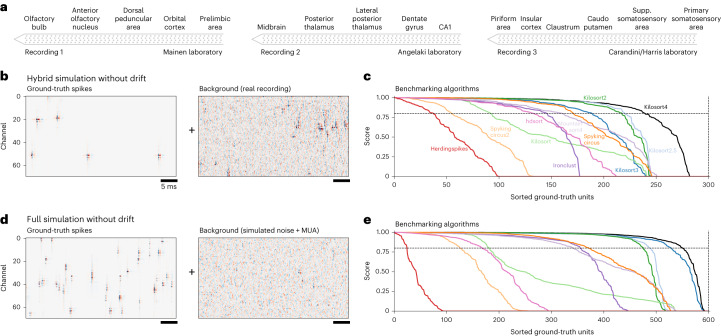
Benchmarks using simulations without drift. **a**, Three low-drift recordings from the IBL from different laboratories and different brain areas. **b**, A short segment of simulated spikes drawn from ground-truth spike waveforms from the recording (left). The real recording was used as background for the simulation (right). **c**, Sorted scores (1 − FP − FN) for each algorithm on 300 ground-truth units pooled across simulations based on the three recordings in **a**. **d**, Same as **b** for a full simulation where the background is generated from 1/f noise and multi-unit activity (MUA) from real experiments. **e**, Same as **c** for the full simulation results, with 600 ground-truth units.

We start in this section with simulations without drift, which are much easier to generate. In this case, we assumed that waveforms are largely stable over a period of time, and we model each spike from the same unit as having the same waveform. Using this assumption, we developed hybrid ground-truth simulations^6,14^ using datasets recorded by the IBL^22^, which specifically had very low levels of drift, as estimated by Kilosort2.5. We chose datasets from a variety of laboratories and spanning different brain areas (Fig. 3a). In hybrid ground-truth approaches, waveforms of the best-isolated units are added as extra spikes over the background provided by the recording, at spatial positions that are vertically offset from where the neuron was originally detected (Fig. 3b). For each ground-truth unit, we matched the units of each algorithm and kept the best match. The matching score was defined as 1 − FP − FN, where FP and FN are the false positive and false negative rates, respectively (Methods). All Kilosort versions except Kilosort1 outperformed all other algorithms, with Kilosort4 performing the best (Fig. 3c).

Next we developed a full simulation, still relying on waveforms from experiments, but generating the spike background from simulated 1/f noise as well as from multi-unit activity using units with small spike norms (Fig. 3d). We found similar performance for all algorithms, both in absolute and relative terms, with Kilosort4 outperforming all other methods. (Fig. 3e).

**Extended Data Fig. 3:**
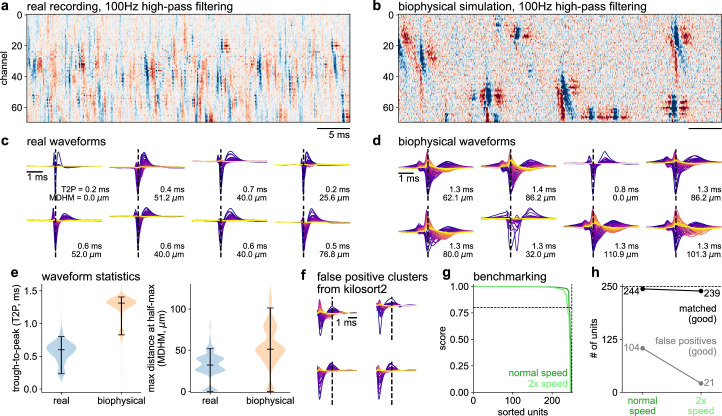
Kilosort false positives from the Spikeinterface paper are due to unrealistically long spike durations. **a**, Segment of a real Neuropixels recording used for comparison. High-pass filtering was applied at 100Hz (rather than 300 Hz) to better illustrate true spike durations. **b**, Same as **a** for a segment of a biophysical simulation. **cd**, Waveforms extracted from the real and simulated recordings respectively. Waveforms were superimposed across channels, using a color code to illustrate channels further from the center (yellow means further). To measure spike duration and spatial extent we used trough-to-peak (T2P) and max-distance at half-max (MDHM) respectively. **e**, Distribution of waveform statistics from the real recording and simulation (n = 292 for real data and n= 250 for simulations, center lines indicate median and error bars indicate 5 and 95 percentiles). **f**, False positive clusters reported by spikeinterface are mostly spikes that were temporally split due to their very long durations. **g**, Benchmarks for original biophysical simulation and a 2x sped up version. **h**, Number of false positives decreases when the simulation is sped up 2x, which makes spike durations more similar to real recordings.

We also considered biophysical simulations as a benchmark but found that existing approaches generate unrealistic waveforms that are outside the distribution of real neurons in the brain (Extended Data Fig. 3a–e). We also found that this mismatch in waveform statistics explained why previous studies using biophysical simulations concluded that Kilosort outputs a large number of false positives^25^ (Extended Data Fig. 3f–h).

### Simulations with realistic drift

**Fig. 4:**
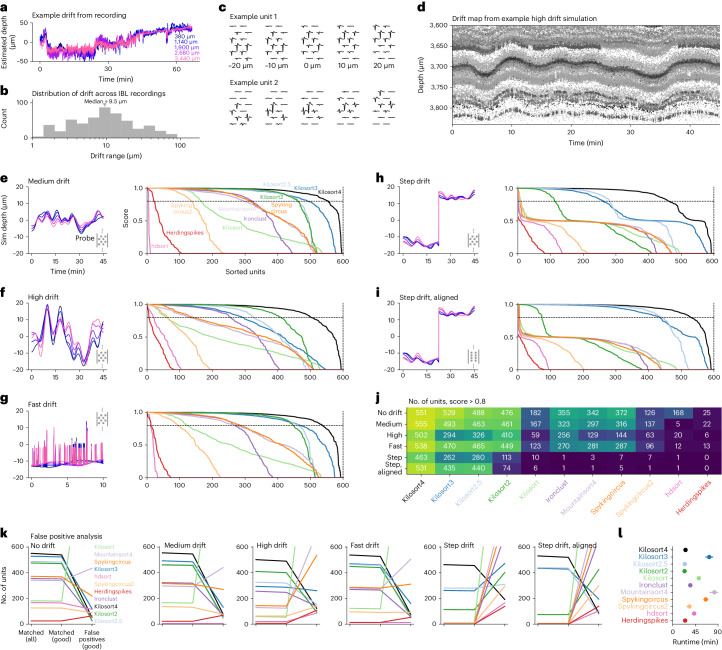
Spike-sorting simulations and benchmarks. **a**, Example drift traces at different depths for a recording with large drift from the IBL dataset. **b**, Distribution of drift ranges across all IBL recordings. Drift range was defined as the difference between the fifth and 95th percentile of the median drift across channels. **c**, Waveforms of example units at multiple drift positions. **d**, Scatter-plot of spike depth versus time, colored by spike amplitude (darker is higher). Spikes were detected from a simulation. **e**–**i**, Accuracy of spike-sorting algorithms on simulations with various drift profiles. Simulated drift traces (left). Sorted accuracies for 600 ground-truth units from each simulation matched to the results of each algorithm (right). The accuracy score is defined as 1 − FP − FN, where FP is the false positive rate and FN is the false negative rate (Methods). **e**, Medium drift. **f**, High drift. **g**, Fast drift (10 min out of 45 min plotted for visibility). **h**, Step drift. **i**, Step drift for a probe with aligned sites. **j**, Summary of units identified at a score > 0.8 across simulations and algorithms. **k**, Number of units matched (same as **j**), matched units that were classified as good (low refractory period violations) and false positive units that were classified as good. Results shown across simulations and algorithms. **l**, Average runtime of each algorithm (mean ± s.e.m., *n* = 6 independent simulations).

**Extended Data Fig. 4:**
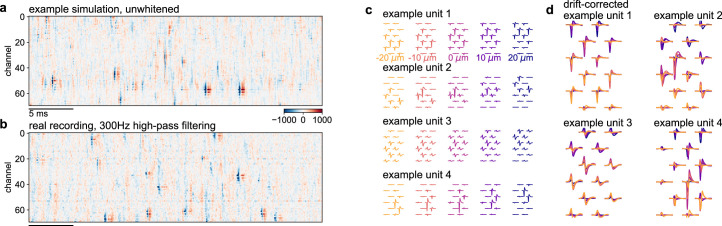
Simulation features. **a**, Example simulation after independent noise was added and “unwhitening" was performed. **b**, Example segment of a real recording high-pass filtered with a cutoff of 300 Hz. **c**, Four additional example units like in Fig. 3c. **d**, The units in **c** after drift correction with the interpolation method from Kilosort 2.5/3/4.

We next developed a set of realistic drifting simulations with a variety of drift patterns. Constructing such a simulation required knowledge of the dense electric fields of a neuron, because different drift levels sample the electric field at different positions. We obtained this knowledge by sampling neurons from recordings with large drift (Fig. 4a) from a public repository of more than 500 Neuropixel recordings from the IBL consortium (Fig. 4b). In this repository, we found 11 recordings with large, continuous drift that spanned over at least 40 μm, which is the spatial repetition period of a Neuropixels probe. We collected two groups of units: one from neurons that were well isolated and had refractory periods and one from multi-unit activity that had refractory period contaminations. The average waveforms at five positions are shown for a few examples (Fig. 4c and Extended Data Fig. 4c,d). To simulate drift, we generated a single average drift trace and additional deviations for each channel to account for heterogeneous drift. Spike trains were generated using shuffled inter-spike intervals from real units. For each simulation, a set of 600 ground-truth neurons were generated in this fashion, with spike norms drawn from a truncated exponential distribution that matched the approximate distribution of norms in real datasets. Another 600 ‘multi-units’ were added with lower norms (Extended Data Fig. 4a). Additional independent noise was added on each channel. The resulting simulation was ‘unwhitened’ across channels using a rotation matrix from real experiments (Extended Data Fig. 4a). The simulations resembled real recordings (Fig. 4d and Extended Data Fig. 4b).

**Extended Data Fig. 5:**
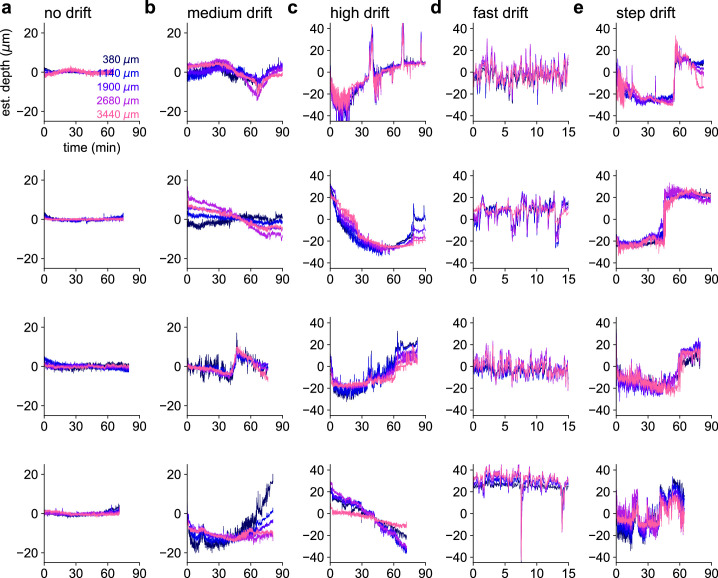
Real drift examples. These are inferred drift traces from the IBL dataset grouped into: **a**, no/small drift, **b**, medium drift, **c**, high drift, **d**, fast drift and **e**, step drift. Note that in many cases different types of drift are combined.

Results for all conditions are shown in Fig. 4e–j and quantified in Fig. 4j. All the algorithms had reasonable run times (Fig. 4l; within 2× the duration of the simulations). The drift conditions we chose were based on patterns of drift identified in the IBL dataset (Extended Data Fig. 5): no drift, medium drift, high drift, fast drift and step drift. We also added an extra condition with horizontally aligned sites for the step drift scenario (such as in Neuropixels2).

### Benchmarks

Kilosort2, 2.5, 3 and 4 again outperformed all other algorithms in all drift conditions. The nearest competing algorithm in performance was IronClust, which accounts for drift in a different way from Kilosort. IronClust generally found ~50% of all units, compared to the 80–90% found by Kilosort4 (Fig. 4j). Many of the algorithms tested did not have explicit drift correction. Some of these (SpyKING CIRCUS and MountainSort4 (refs. ^2,3^)) matched the IronClust performance at no drift, medium and fast drift, but their performance deteriorated drastically with higher drift. Among all algorithms with explicit drift correction (Kilosort2.5, 3 and 4), Kilosort4 consistently performed better due to its improved clustering algorithm and in some cases performed much better (on the step drift conditions).

**Extended Data Fig. 6:**
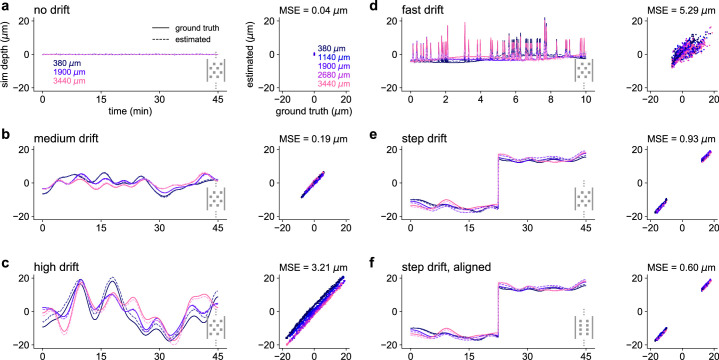
Recovered drift traces from simulations. **a-f**, Ground truth simulated drift + the drift identified by Kilosort4. (Left) Estimated and true drift traces. (Right) Scatter plot of estimated and true drift traces.

**Extended Data Fig. 7:**
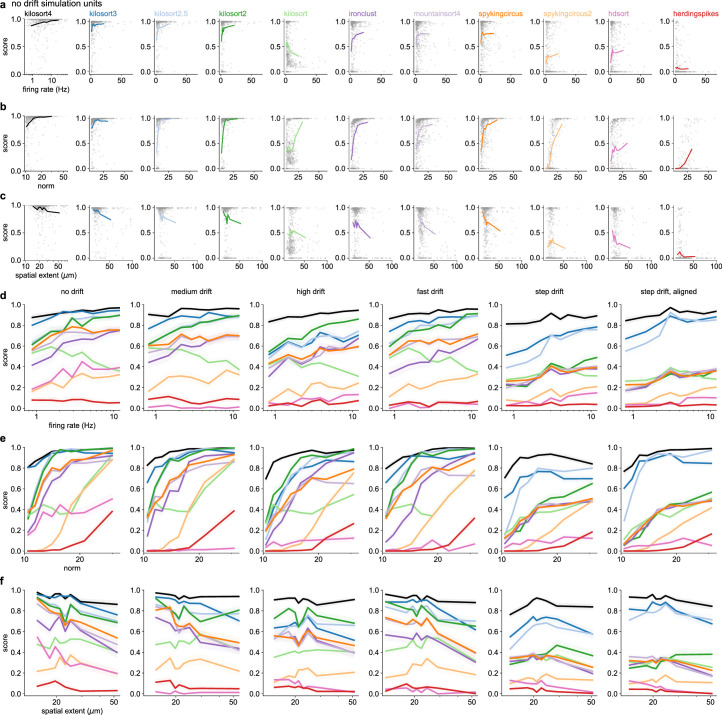
Accuracy as a function of firing rate, amplitude/norm and spatial extent. **a-c**, Scatter plots of unit properties (firing rate, norm, spatial extent respectively) versus accuracy, for the no drift simulation. Lines show the average accuracy in bins of equal numbers of points. **d-f**, Average accuracy curves for all types of simulations and all unit properties (firing rate, norm, spatial extent respectively).

We also tested how well the drift amplitudes were identified by the drift detection algorithm from Kilosort2.5 (in the Kilosort4 implementation) and found good performance in all cases, except for the fast drift condition where the timescale of drift was faster than the 2-s bin size used for drift correction (Extended Data Fig. 6). Much smaller bin sizes cannot be used for drift estimation, as a minimum number of spike samples is required. Nonetheless, the results show that Kilosort still performed well in this case, likely due to the robustness of the clustering algorithms. Finally, we calculated the performance of the algorithms as a function of the ground-truth firing rates, spike norm and spatial extents (Extended Data Fig. 7). The dependence of Kilosort4 on these variables was minimal; however, some of the other algorithms had a strong dependence on spike norm, which could not be improved by lowering spike detection thresholds. Also, many algorithms performed poorly when the waveforms had a large spatial extent as opposed to having their electrical fields concentrated on just a few channels.

Next, we performed a false positive analysis to see whether the high number of units correctly identified by Kilosort4 comes at the cost of many false positive units (Fig. 4k). For this analysis, we only considered ‘good’ units as putative candidates (units with low refractory period violations), as those are the units that users would consider further. To maintain consistency, we defined ‘good’ units in the same way for all algorithms. First, we noticed that the matched (good) units were generally very similar to the matched (all) units. Second, we noticed that across simulations Kilosort4 had similar numbers of false positive units compared to the other algorithms, which were generally in the range of 50–100 units. These likely correspond to pieces of ground-truth units that were not matched at the 0.8 threshold that we imposed on the scores. Thus, the high performance of Kilosort4 and other Kilosort versions does not come at the cost of high false positive unit rates.

### Ablation results for Kilosort

In this section, we investigate the effect on performance of different steps in Kilosort. To start, we can gain insight by comparing certain pairs of Kilosort versions. Kilosort2 and 2.5 only differ in their drift-correction strategy and perform similarly on most simulations except for the step drift conditions, where Kilosort2 performs more poorly. This is due to the drift-tracking approach of Kilosort2, which needs a continuous distribution of drift positions. We can also compare Kilosort2.5, 3 and 4, which have the same drift-correction strategy and the same template deconvolution strategy but differ in the clustering algorithm. The graph-based clustering from Kilosort4 helps across all drift conditions.

**Fig. 5:**
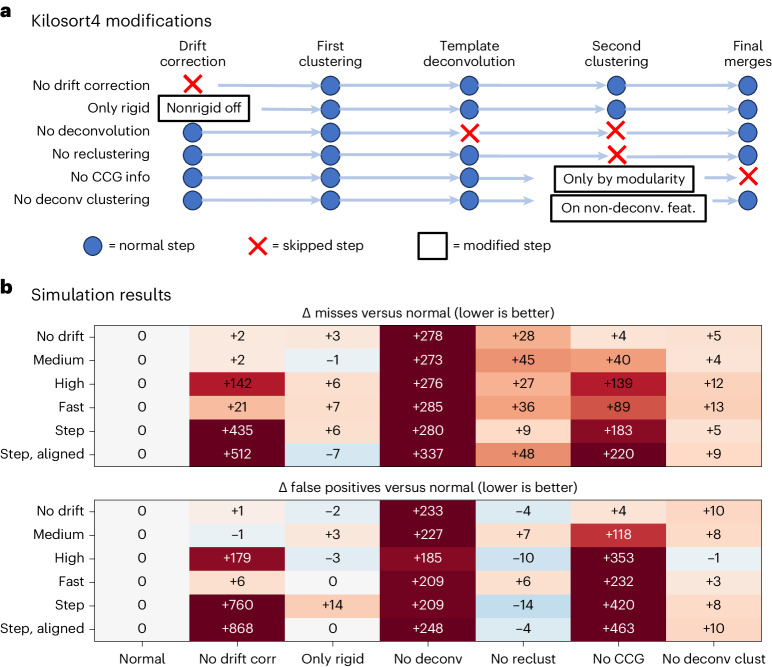
Ablation results. **a**, We tested six different ablations across all simulation conditions: no drift correction, rigid motion correction only, no template deconvolution, no reclustering after template deconvolution, no cross-correlogram (CCG) information in deciding splits and merges and no feature deconvolution for the final clustering step. **b**, Simulation results quantified as changes in the number of misses (top) and changes in the number of false positives (bottom).

We also performed an ablation study on Kilosort4 by disabling certain algorithmic steps (Fig. 5a). We tested the performance of six different variations of Kilosort4 across all simulations and evaluated misses as well as false positives (Fig. 5b). Some steps had strong effects on performance: drift correction, deconvolution and cross-correlogram-based merges/splits. The reclustering step after template deconvolution had a smaller but consistent effect, as turning it off resulted in more misses, but relatively no change in false positives. The least change was observed by turning off nonrigid motion correction, which was surprising as the simulations contained a substantial fraction of nonrigid drift. Similarly, turning off the deconvolution for feature extraction had only a small, though consistent effect, despite the substantial effect it seems to have on the extracted features (Fig. 1h,i). A likely explanation for both these effects may be that some steps in Kilosort4 can redundantly fix problems left over by the other steps. In this case, the clustering algorithm may itself be sufficiently robust to work on non-deconvolved features and without fully nonrigid motion correction; however, we cannot rule out that in some recording scenarios these steps are more important.

## Discussion

Here we described Kilosort, a computational framework for spike-sorting electrophysiological data. All versions of Kilosort have been developed primarily on Neuropixels data; however, as Kilosort adapts to the data statistics, it has been used widely on other types of probes and other recording methods. We also tested Kilosort4 on two publicly available datasets recorded with either a 64-channel linear probe^26^ or a 128-channel tetrode array^27^ and found that Kilosort4 returned good results in both cases (Extended Data Fig. 2). Kilosort4 should substantially reduce the amount of manual curation required for different types of probes and recordings, though we encourage users to continue checking the quality of their results in Phy^14^.

Some types of data do require special consideration. For example, some data cannot be drift-corrected effectively due to either lacking a well-defined geometry (tetrodes) or due to the vertical spacing between electrodes being too high (more than 40 μm). This consideration also applies to data from single electrodes such as in a Utah array. Data from retinal arrays do not require drift correction and may be processed through Kilosort4 but may require large amounts of GPU RAM for arrays with thousands of electrodes and thus would be better split into multiple sections and processed separately. Another special type of recording comes from chronic experiments over multiple days, potentially separated by long intervals. While we have not explicitly tested such recordings here, the benchmark results for the step drift simulation are encouraging because this simulation qualitatively matches changes we have seen chronically with implanted Neuropixels2 electrodes^13^.

The problem of identifying neurons from extracellular recordings has a long history in neuroscience. The substantial progress seen in the past several years stems from multiple simultaneous developments: engineering of better devices (Neuropixels and others), better algorithms (Kilosort and others), improved visualizations of spike-sorting results (Phy) and multiple rounds of user feedback provided by a quickly expanding community. Computational requirements have sometimes influenced the design of new probes, such as the aligned sites and reduced vertical spacing of Neuropixels2, which were motivated by the need for better drift correction. Such computational considerations will hopefully continue to influence the development of future devices to increase the quality and quantity of neurons recovered by spike sorting.

## Methods

The Kilosort4 code library is implemented in Python 3 (ref. ^28^) using pytorch, numpy, scipy, scikit-learn, faiss-cpu, numba and tqdm^29–36^. The GUI additionally uses PyQt and pyqtgraph^37^. The figures were made using matplotlib and jupyter-notebook^38,39^. Kilosort2, 2.5 and 3 were implemented in MATLAB.

To be able to process the large amount of data from modern electrophysiology, all versions of Kilosort were implemented on the GPU. Kilosort4 is the first version fully implemented in Python, using the pytorch package for all its functionality, thus making the old CUDA functions obsolete^28,31^. Pytorch allows the user to switch to a CPU back end, which may be sufficiently fast for testing on small amounts of data but is not recommended for large-scale data. All versions of Kilosort take as input a binary data file, and output a set of ‘.npy’ files that can be used for visualization in Phy^14^. To set up a Kilosort4 run, we built a pyqtgraph GUI that replicates the functionality of the MATLAB GUI and can assist users in debugging due to the display of several diagnostic plots and summary statistics^37^ (Extended Data Fig. 2).

We demonstrate the Kilosort4 method step-by-step in Figs. 1 and 2. In Fig. 1 an electrophysiological recording from N. Steinmetz was used (‘Single Phase 3’ (ref. ^16^); https://figshare.com/articles/_Single_Phase3_Neuropixels_Dataset/7666892). In Fig. 2 an electrophysiological recording from the IBL was used (ID 6f6d2c8e-28be-49f4-ae4d-06be2d3148c1)^22^. In Fig. 3a–c, three recordings with very little drift were chosen to create hybrid ground-truth simulations: 3f6e25ae-c007-4dc3-aa77-450fd5705046, fe380793-8035-414e-b000-09bfe5ece92a and 4ddb8a95-788b-48d0-8a0a-66c7c796da96. In Figs. 3d and 4, drifting waveforms were extracted from high-drift IBL recordings:

- 671c7ea7-6726-4fbe-adeb-f89c2c8e489b
- eacc49a9-f3a1-49f1-b87f-0972f90ee837
- 0c828385-6dd6-4842-a702-c5075f5f5e81
- 32d27583-56aa-4510-bc03-669036edad20
- 58c4bf97-ec3b-45b4-9db4-d5d9515d5b00
- cea755db-4eee-4138-bdd6-fc23a572f5a1
- 68775ca0-b056-48d5-b6ae-a4c2a76ae48f
- d57df551-6dcb-4242-9c72-b806cff5613a
- cde63527-7f5a-4cc3-8ac2-215d82e7da26
- fc14c0d6-51cf-48ba-b326-56ed5a9420c3
- 4ddb8a95-788b-48d0-8a0a-66c7c796da96.

All these recordings were performed with a Neuropixels1.0 probe, which has 384 sites organized in rows of two with a vertical spacing of 20 μm, a horizontal spacing of 32 μm. Due to the staggered design (16-μm horizontal offset between consecutive rows), the spatial repetition period of this probe is 40 μm. For loading data, provided scripts were adapted (https://github.com/int-brain-lab/mtscomp).

### GUI

We developed a GUI to facilitate the user interaction with Kilosort4. This interface was built using pyqtgraph, which itself uses PyQt^37,40^, and it replicates the MATLAB GUI that was originally built for Kilosort2 by N. Steinmetz. The GUI allows the user to select a data file, a configuration file for the probe and set the most important parameters manually. In addition, a probe file can be constructed directly in the GUI. After loading the data and configuration file, the GUI displays a short segment of the data, which can be used to determine whether the configuration was correct. Typical mistakes are easy to identify. For example, if the total number of channels is incorrect, then the data will seem to be diagonally ‘streaked’ because multi-channel patterns will be offset by one or two extra samples on each consecutive channel. Another typical problem is having an incorrect order of channels, in which case the user will see clear single-channel but no multi-channel waveforms. Finally, the GUI can produce several plots during runs, which can be used to diagnose drift correction and the overall spike rates of the recording.

### Algorithms for Kilosort4

In the next few sections, we describe the algorithmic steps in Kilosort4. Some of these steps are inherited or evolved from previous versions. For clarity, we describe each of the steps exactly as they are currently used in Kilosort4. If a previous version of Kilosort is different, we clearly indicate the difference. We also describe separately in the Supplementary Information the algorithms not used in Kilosort4 but used in previous versions.

Many of the processing operations are performed on a per-batch basis. The default batch size was *N*_T_ = 60,000 in v.4 and it was *N*_T_ = 65,536 in v.2, 2.5 and 3 and *N*_T_ = 32,768 in v.1. The increase in batch size in Kilosort2 was designed to allow better per-batch estimation of drift properties. Due to the per-batch application of temporal operations, we require special considerations at batch boundaries. Every batch of data is loaded with left and right padding of *n*_t_ additional time points on each side (*n*_t_ = 61 by default). On the first batch, the left pad consists of the first data sample repeated *n*_t_ times. The last batch is typically less than a full batch size of *N*_T_. For consistency, we pad this batch to the full *N*_T_ size using the repeated last value in the data. The batch size as well as the padding are user-modifiable.

The clustering in Kilosort3 and Kilosort4 is conducted in small sections of the probe (for example, 40 μm for Neuropixels1), but including information from nearby channels and including spikes extracted at all time points.

### Preprocessing

Our standard preprocessing pipeline includes a sequence of operations: common average referencing (CAR), temporal filtering, channel whitening and drift correction. These steps are applied in sequence; drift correction uses data that have undergone CAR, temporally filtered and channel-whitened. In Kilosort4, all these steps are performed on demand whenever a batch of data is needed. In all previous versions, the preprocessing of the entire data was conducted first and the preprocessed data were stored in a separate binary file. Drift correction was introduced in Kilosort2.5.

#### Data formats

The standard data format for Kilosort is a flat binary file with a default data type of ‘int16’. If the data type is different, the user needs to specify one of ‘uint16’, ‘int32’ or ‘float32’. If the file format is different, the user must either convert the data to binary using SpikeInterface^25^ (preferable, for compatibility with Phy^14^ and faster speed) or use our SpikeInterface wrapper to load data into Kilosort without doing the conversion. We provide an example notebook to illustrate the data format conversion using SpikeInterface^25^ (https://github.com/MouseLand/Kilosort/blob/main/docs/tutorials/load_data.ipynb), which allows for compatibility with several more formats, such as ‘nwb’, ‘open-ephys’, ‘blackrock’, ‘neuralynx’ and ‘intan’.

#### CAR

The first operations applied to data are to remove the mean across time for each batch, followed by removing the median across channels (CAR). The CAR can substantially reduce the impact of artifacts coming from remote sources such as room noise or optogenetics. The CAR must be applied before the other filtering and whitening operations, so that large artifacts do not ‘leak’ into other data samples.

#### Temporal filtering

This is a per-channel filtering operation that defaults to a high-pass filter at 300 Hz. Bandpass filtering is typically performed using IIR filters, for example with Butterworth coefficients. Butterworth filters have some desirable properties in the frequency space, but their implementation on the GPU is slow. To accelerate it, we switch to using an FIR filter that simulates the Butterworth filter and we perform the FIR operation in FFT space taking advantage of the convolution theorem. To get the impulse response of a Butterworth filter, we simply filter a vector of size *N*_T_ with all zeros and a single 1 value at position floor(*N*_T_/2) (0-based indexing).

#### Channel whitening

While temporal filtering reduces time-lagged correlations coming from background electrical activity, it does not reduce across-channel correlations. To reduce the impact of local sources, such as spikes from 100–1,000 μm away from the probe, we perform channel whitening in local neighborhoods of channels. A separate whitening vector is estimated for each channel based on its nearest 32 channels using the so-called ZCA (zero-phase component analysis) transform^41^. ZCA is the data-whitening transformation that is closest in Euclidean norm to the original data. For an *N* × *T* matrix A, the ZCA transform matrix *W* is found by inverting the covariance matrix, using epsilon-smoothing of the singular values:

$$\begin{array}{l} \qquad\,\, {C}={\rm{cov}}(A)\\ U,S,V={\rm{svd}}(C) \\ \qquad \, {W}=U{(S+\epsilon I)}^{-\frac{1}{2}}{U}^{T} \\ \end{array}$$

The local whitening matrix *W* is calculated separately for each channel and its neighborhood of 32 channels, and only the whitening vector corresponding to that channel is kept and embedded into a full-size *N*_chan_ × *N*_chan_ matrix. This is preferable to directly calculating a grand *N*_chan_ × *N*_chan_ whitening matrix because it reduces the number of whitening coefficients to 32 × *N*_chan_ instead of *N*_chan_ × *N*_chan_, which prevents overfitting in the limit of a large *N*_chan_. The number of neighborhood channels is user-modifiable and may need to be increased for ultra-dense probes, such as the Neuropixels Ultra^42^.

#### Drift correction

Drift correction is a complex preprocessing step that was described in detail previously^13^. Here we describe only a few small modifications in Kilosort4. The drift correction process can be separated into drift estimation and data alignment. In Kilosort4, drift estimation is performed in advance, whereas data alignment is performed on demand, along with the other preprocessing operations. Drift estimation includes a step of spike detection, which uses a set of predefined, ‘simple’ templates to detect multi-channel spikes. In Kilosort2.5 and 3, these predefined templates were constrained to be negative-going spikes, whereas in Kilosort4 we consider both positive and negative-going spikes using pairs of inverted templates (for fast computation). Another modification in Kilosort4 is the use of linear interpolation for sampling the drift traces at every channel, in place of the ‘Makima’ method used in previous versions.

As data alignment is a linear operation performed with a Gaussian kriging kernel, it can be combined with channel whitening, which is also a linear operation. In practical terms, the two *N*_chan_ × *N*_chan_ matrix multiplications are combined into one, thus further accelerating the computation.

### Template deconvolution

Template deconvolution is the process of using a set of waveform templates matched to the data to detect spikes and extract their features, even when they overlap other spikes on the same channels and at the same time points. Template deconvolution can be seen as replacing the spike detection step in a classical spike-sorting pipeline. The goal in Kilosort4 is to extract all the spikes above a certain waveform norm and calculate their spike features in a way that discards the contribution of nearby overlapping spikes. Template deconvolution improves on classical spike detection in several ways:

1. The detection of the spikes is performed by template matching, which is a more effective way of detecting spikes compared to threshold crossings, because it uses templates that represent the multi-channel spikes of the neurons being matched.
2. Spikes that overlap in time and channels can be detected and extracted as separate events due to the use of an iterative matching pursuit. Classical methods require an ‘interdiction’ area in time and channels around each detected spike where a second spike detection is disallowed, to prevent double detections of the same spike.
3. The features extracted for each spike can be decontaminated from other overlapping spikes, due to the use of a generative or reconstructive model. As described below, these features are robust to imperfect templates, because the templates are only used for detection and for subtracting other spikes from the background, and they are not used to compute the features of the detected spike itself.

#### Template learning

To perform template deconvolution, a set of templates must be learned that can match all the detectable spikes on the probe. In previous Kilosort versions (1, 2 and 2.5), special care was taken to ensure that these templates match neural waveforms on a one-to-one basis. This was necessary because relatively few additional merges and splits were performed after template deconvolution. In Kilosort3 and 4, the templates do not need to match single neurons because the features extracted by template deconvolution are clustered again using more-refined clustering algorithms; however, it is important that every spike in the raw data has some template to match to.

To build a set of templates, we performed clustering on a set of spikes identified using a set of simple spike templates. This initial spike detection step is equivalent to the spike detection performed in Kilosort2.5 for drift correction. The simple templates are defined by all possible combinations of (1) a spatial position in two dimensions; (2) a single-channel waveform shape; and (3) a spatial size. The spatial positions need not be coincident with actual probe channels and we choose them to upsample the channel densities by a factor of 2 in each dimension. For a Neuropixels1 probe, this corresponds to 1,536 positions. The single-channel waveform shapes are obtained by *k*-means clustering of single-channel spikes, either from a pre-existing dataset (IBL dataset) or from spikes detected by threshold crossings in the data, and we defaulted to six such waveforms. Finally, the spatial sizes (five by default) define the envelope of an isotropic Gaussian centered on the spatial position of the template, which is used as per-channel amplitudes. In total, a set of 46,080 simple templates are used for a Neuropixels1 probe corresponding to all possible combinations of spike shapes, spike sizes and two-dimensional spike positions (for more details see ref. ^13^). The spatial footprints are explicitly precomputed for all positions and all spatial sizes. The templates are effectively normalized to unit norm by separately normalizing the per-channel waveform templates and the spatial footprints. As the simple templates are unit norm, their variance explained (*V*_explained_) at each time point can be easily calculated as the dot product with the data, squared:

$$\begin{array}{l}{V}_{{{{\rm{explained}}}}}\,=\parallel D{\parallel }^{2}-{\min }_{x}\parallel D-xW{\parallel }^{2}\\\qquad\qquad \,=\parallel D{\parallel }^{2}-\parallel D-({W}^{T}D)W{\parallel }^{2}\\\qquad\qquad \,={({W}^{T}D)}^{2}\end{array}$$

where *W* is the unit-norm simple template, *D* are the data over a particular set of channels and time points and *x* is the best-matching scalar norm that the template needs to be multiplied by to match the data.

The dot products between each of these templates and the data at each time point can be performed efficiently in the following order: (1) temporal convolution of each data channel with each of the six single-channel waveforms; and (2) per time point matrix multiplication with a set of weights corresponding to all positions and all spatial sizes. Once the dot products are calculated in this manner, the largest variance explained value is kept at each spatial position of each template. For a Neuropixels probe, this is a matrix of size 1,536 × *N*_T_ (batch size). The goal of this spike detection step is to find localized peaks in this matrix, which must be local maxima in a neighborhood of time points (± 20) and spatial positions (100 nearest positions). The relatively large neighborhood size ensures that no spike is detected twice, but prevents many overlapping spikes from being detected (typically about 50% of spikes go undetected); however, the missing spikes are not a concern for the purpose of template learning, as it is extremely unlikely that all the spikes from a neuron will be consistently missed by this procedure.

Once the spikes are detected, we extract PC features in the ten nearest channels to each detection. We use a set of six PCs that are found either from a pre-existing dataset (IBL dataset) or from spikes detected by threshold crossings. For each spike, an *xy* position on the probe is computed based on the center of mass across channels of the spike’s projection on the best-matching single-channel template (same as in Kilosort2.5). We assign all spikes in 40-μm bins according to their vertical position and embed all spikes detected in the same bin to the same set of channels (which is usually more than ten channels due to differences between spike positions). Finally, the embedded PC features are clustered according to the same graph-based clustering algorithm we describe below, using only the merging criterion of the bimodal regression axis and not using the cross-correlation based criterion. In Kilosort3, the same procedure is applied but the clustering algorithm is recursive pursuit. After clustering each 40-μm section of the probe, the centroids are multiplied back from PC space into spatiotemporal waveforms and pooled together across the probe.

Templates from the same neuron may be detected multiple times, either on the same 40-μm section or in nearby sections. This is not inherently a problem because each neuron can have multiple templates; however, it can become a problem if these multiple templates are not aligned to each other, because then, spikes from the same neuron will be detected at different temporal positions, which changes their PC feature distribution. In addition, having many templates makes the spike-detection step memory and compute inefficient. A solution to both these problems is to merge templates that have a high correlation with each other and similar means, where the correlation is maximized across possible timelags. In addition, we temporally align all templates based on their maximal correlation with the same six prototypical single-channel waveforms described above. Note that this merging step may result in the opposite scenario of having one template for multiple neurons. This is also not a problem, because templates are only merged when they have a high correlation and thus the same average template can successfully match the shape of multiple neurons.

#### Spike detection with learned templates and matching pursuit

Once a set of templates is learned, they can be used for template matching similar to the simple templates described above. The main difference is that instead of allowing for an arbitrary scaling factor *x*, we require that matches use the average norm of the template it was found with. The *V*_explained_ of learned template *W* of some data *D* thus becomes:

$$\begin{array}{ll}{V}_{{{{\rm{explained}}}}}\,=\parallel D{\parallel }^{2}-\parallel D-{x}_{W}W{\parallel }^{2}\\\qquad\qquad \,=2{x}_{W}{W}^{T}D-{x}_{W}^{2}\\ \end{array}$$

Like before, this quantity only requires the calculation of *W*^*T*^*D*, which can be performed convolutionally for each template. In practice, we represent templates using a three-rank approximation, factorized over channels and time, which speeds up the convolutions dramatically^6^. We first multiply the data with the channel weights for each rank and convolve the resulting traces with the temporal components. The three-rank approximation captures nearly the entire waveform variance in all cases^6^ and also helps to denoise templates calculated from relatively few spikes.

To extract overlapping spikes, we must detect spikes iteratively over the same portion of data and subtract off from the data those parts attributed to spike detections. This subtraction allows for another pass of detections to be performed, which can detect other spikes left over and yet unsubtracted. This procedure is called matching pursuit^43^ and is fundamentally a sequential process; to detect another spike, one must first subtract off the contributions of spikes detected before; however, we can parallelize this step, thus making it suitable for GPU processing by observing that the subtraction of a single spike results in highly localized changes to the data, which cannot affect the calculated spike norms far from the position of that subtracted spike. Thus, we can detect and subtract multiple spikes in one round as long as they are far enough from each other. Upon calculating a matrix of variance explained for each template at each time point, we detect peaks in this matrix that are local maxima over local neighborhoods in time ± *n*_t_ time samples and across all channels. After detection, the optimal norm for each spike is calculated and its contribution from the data is subtracted off. To avoid recalculating the dot products of templates at all time points, the contribution of the subtracted spikes to the dot products is directly updated locally using a set of precomputed dot products between templates, at all possible timelags. This detection and subtraction process is repeated for 50 rounds, with later rounds being much faster due to the increasingly smaller number of spikes left to extract.

#### Extracting PC features with background subtraction

The final step in template deconvolution is to extract features from the data to be used by the clustering algorithm. One possibility would be to directly extract PC features from the preprocessed data at the spike detection times (Fig. 1h); however, this results in contamination with background spikes. A better option is to first subtract the effect of other spikes, as we know from the matching pursuit step how much these other spikes contribute (Fig. 1e). To do this computation efficiently, we first extract PC features from the residual (Fig. 1f), and then add back to these features the contribution of the template that was used to extract the spike. The contribution of each template in PC space is precomputed for faster processing.

### Graph-based clustering

The new clustering algorithm in Kilosort4 uses graph-based algorithms. This class of algorithms relies entirely on the graph constructed by finding the nearest neighbors to each data point. There are several steps:

1. Neighbor finding with subsampling
2. Iterative neighbor reassignment
3. Hierarchical linkage tree.

#### Neighbor finding with subsampling

Many frameworks for fast neighbor finding exist and we tested many of them for spike-sorting data. In the end, the brute force implementation from the faiss framework^30^ outperformed other approaches in speed on modern multi-core computers for the range of data points that we need to search over (10,000–100,000) and the number of data points that we need to find neighbors for (100,000–1,000,000).

#### Iterative neighbor assignment

Clustering algorithms based on graphs typically optimize a cost function such as the modularity cost function. We review this approach first, before describing our new approach. Following ref. ^19^, the modularity cost function is defined by

$${{{\mathcal{H}}}}=\frac{1}{2m}\mathop{\sum}\limits_{c}\left({e}_{c}-\gamma \frac{{K}_{c}^{2}}{2m}\right)$$

where *m* is the total number of edges in the graph, *e*_*c*_ is the number of edges in community *c*, *K*_*c*_ is the sum of degrees in community *c* and *γ* is a ‘resolution’ parameter that controls the number of clusters. The $\frac{{K}_{c}^{2}}{2m}$ can be interpreted as the expected number of edges in community *c* from a null model with the same node degrees as the data but otherwise random graph connections.

Specialized optimization algorithms exist to maximize the modularity cost function by moving nodes between communities and performing merges when the node reassignment converges^20^. Additionally, splitting steps and other optimizations were recently introduced, which improve the results of the algorithm and its speed^19^. These algorithms are effective for many types of data, yet have a substantial failure mode for spike-sorting data: they have difficulty clustering data with very different number of points per cluster. In practice, for our clustering problems, there are often very large clusters of up to 100,000 points together with clusters with many fewer (<1,000) points. A low-resolution parameter *γ* can keep the large cluster in one piece, but also merges the small clusters into larger clusters. Conversely, high-resolution parameters may return the small clusters as individual clusters, but can split the large cluster into very many (hundreds) of pieces. The oversplitting is not inherently a bad property as we will perform merges on these clusters anyway, but the large number of pieces returned for the large clusters means that many correct merging decisions must be made, which is in itself a very difficult optimization problem. In addition, running the Louvain/Leiden algorithms with large resolution parameters may somewhat reduce the effectiveness of the algorithm, as the community penalty $\gamma \frac{{K}_{c}^{2}}{2m}$ only has a null model interpretation for *γ* = 1.

To improve on these algorithms, we started from the observation that local minima of the neighbor reassignment step have some desirable properties. These local minima arise because the neighbor reassignment step monotonically improves the modularity cost function by greedily moving nodes to new clusters if that improves the modularity score. This step converges after a while, because no more clusters can be moved. This is, however, a local minimum of the optimization, and the modularity can often be further increased by making merges between clusters. Unlike the node reassignment, which consists of small local moves, the merging between clusters is a global move in the cost function and can thus escape the local minimum. Algorithms such as Leiden/Louvain take advantage of such global merges by applying the node reassignment step again on a new graph made by aggregating all the points into their clusters when the local minimum is reached.

Our observation was that the local minima themselves can consist of good clustering (Fig. 2b). We initialize the algorithm with 200 clusters found by the *k*-means++ algorithm, a popular initialization choice for clustering^44^. The node reassignment algorithm for the modularity cost function with *γ* = 1 is run for a fixed number of iterations (typically sufficient for convergence). The converged partitioning of the data is then used as a clustering result. Especially relevant to the next step, the algorithm almost never made incorrect merges and instead, output some clusters oversplit. This bias toward oversplitting is important because it allows us to correct the mistakes of the algorithm by making correct merge decisions, which is much easier than finding the correct split in a cluster.

We also found that clusters that were oversplit generally had a reason to be oversplit; the separate pieces identified by the algorithm were in fact sufficiently different to create a local minimum in the cluster assignments. This is a common problem in spike-sorting data, where nonlinear changes in the waveform can result in clusters that seem bimodal in Euclidian space. An extreme example of this effect is due to abrupt drifts of the probe changing the sampling of the waveforms by a non-integer multiple of the probe period. Even after drift correction, waveforms sampled at the two different positions will be much more similar to other waveforms from the same position than they are to waveforms sampled at the other position (Extended Data Fig. 4b). As a consequence, many algorithms return such units oversplit into two halves, as can be clearly seen in the benchmark results for the step drift condition, where many units are identified with exactly a 0.5 score, which corresponds to 50% of the spikes identified.

#### Hierarchical merging tree

To perform merges, we could take two strategies: (1) a brute force approach in which we check all pairs of clusters for merges or at least the ones with high waveform correlation; and (2) a directed approach, where we use the structure of the data to tell us which merges to check. We use both, starting with the second one to reduce the number of clusters and thus reduce the number of brute force checks we need to make later.

For the directed approach, we construct a hierarchical merging tree based on the modularity cost function. The leaves of this tree consist of the clusters identified at the previous step. For each pair of clusters *i*,*j*, we aggregate the neighbors and node degrees, similar to the Leiden/Louvain algorithms, thus resulting in a full matrix *K* of size *n*_k_ by *n*_k_, where *n*_k_ is the number of clusters and where *K*_*ij*_ is the number of edges between clusters *i*,*j*, while *K*_*ii*_ is the number of internal edges. Additionally, a variable *k*_*i*_ holds the aggregated degree of each cluster *i*. The linkage tree is constructed by varying the resolution parameter *γ* in the modularity cost function from *∞* down to 0. As *γ* decreases, merges of two clusters start to increase the modularity cost function. Specifically, a pair of clusters gets merged when the modularity ${{{{\mathcal{H}}}}}_{2}$ after merging equals the modularity ${{{{\mathcal{H}}}}}_{1}$ before merging, where:

$$\begin{array}{rc}{{{{\mathcal{H}}}}}_{1}&=\left({K}_{ii}-\gamma \frac{{k}_{i}^{2}}{2m}\right)+\left({K}_{jj}-\gamma \frac{{k}_{j}^{2}}{2m}\right)+{{{\rm{constant}}}}\\ {{{{\mathcal{H}}}}}_{2}&=\left({K}_{ij}+{K}_{ii}+{K}_{jj}-\gamma \frac{{({k}_{i}+{k}_{j})}^{2}}{2m}\right)+{{{\rm{constant}}}}\end{array}$$

Setting $${{{{\mathcal{H}}}}}_{2}={{{{\mathcal{H}}}}}_{1}$$ yields:

$$\begin{array}{ll}{{{{\mathcal{H}}}}}_{2}-{{{{\mathcal{H}}}}}_{1}\,={K}_{ij}-{\hat{\gamma }}_{ij}\frac{{k}_{i}{k}_{j}}{2m}=0\\\qquad\quad {\hat{\gamma }}_{ij}\,=\frac{2m{K}_{ij}}{{k}_{i}{k}_{j}}\end{array}$$

In other words, a pair of clusters *i*, *j* should be merged when *γ* reaches a value of 2*mK*_*ij*_/(*k*_*i*_*k*_*j*_). After merging, the matrix *K* and vector *k* can be recomputed with the two clusters *i*, *j* becoming aggregated into one. Note that a merging decision does not change the $\hat{\gamma }$ for other pairs of clusters, and it cannot result in a higher $\hat{\gamma }$ than the current ${\hat{\gamma }}_{ij}$. This can be shown by reductio ad absurdum; if the merged *i*,*j* cluster had a higher $\hat{\gamma }$ with another cluster *l*, it would imply that one of the original clusters *i* or *j* had a higher ${\hat{\gamma }}_{il}$ or ${\hat{\gamma }}_{jl}$, and thus it should have been merged a priori. The monotonic property of ${\hat{\gamma }}_{ij}$ ensures that a well-defined merging tree exists, with a strictly decreasing sequence of $\hat{\gamma }$ for increasingly higher merges in the tree. Empirically, we have found that the resulting merging tree is very useful for making merge/split decisions.

### Split/merge criteria

With the tree constructed, we next move down the tree starting from the top and make individual merge/split decisions at every node. If a node is not being split, then the splits below that node are no longer checked. We use two splitting criteria: (1) the bimodality of the data projection along the regression axis between the two clusters and (2) the degree of refractoriness of the cross-correlogram. These two criteria tend to be the ones most used by human curators performing spike sorting. If the pair of units has a refractory cross-correlogram, then the split is always performed. If the cross-correlogram is not refractory, then the split is performed if and only if the projection along the regression axis is bimodal. In addition, splits below a predefined small modularity threshold (0.2) are always accepted to prevent cases where the top nodes are not split (though we never observed such cases).

#### Bimodality of regression axis

Consider a set of spike features **x**_*k*_ with associated labels *y*_*k*_ ∈ {−1, 1}, where −1 indicates the first cluster and 1 indicates the second cluster. A regression axis $\hat{{{{\bf{u}}}}}$ can be obtained by minimizing:

$$\begin{array}{rc}\hat{{{{\bf{u}}}}}&={{{{\rm{argmin}}}}}_{u}\displaystyle\mathop{\sum}\limits_{k}{\left({{{{\bf{u}}}}}^{T}{{{{\bf{x}}}}}_{k}-{y}_{k}\right)}^{2}\end{array}$$

This regression problem becomes highly unbalanced when one of the clusters has many more points than the other. We therefore add a set of weights *w*_−1_ = *n*_2_/(*n*_1_ + *n*_2_), *w*_+1_ = *n*_1_/(*n*_1_ + *n*_2_), where *n*_1_, *n*_2_ are the number of spikes in the first and second cluster.

$$\begin{array}{rc}\hat{{{{\bf{u}}}}}&={{{{\rm{argmin}}}}}_{u}\displaystyle\mathop{\sum}\limits_{k}{w}_{{y}_{k}}{\left({{{{\bf{u}}}}}^{T}{{{{\bf{x}}}}}_{k}-{y}_{k}\right)}^{2}\end{array}$$

This weighted regression problem can be solved in the usual fashion. Finally, we use the $\hat{{{{\bf{u}}}}}$ axis to estimate how well separated the clusters are by projecting ${x}_{\mathrm{proj}}={\hat{{{{\bf{u}}}}}}^{T}{{{{\bf{x}}}}}_{k}$. The density of the projections is estimated nonparametrically. The projections are binned in 400 bins linearly-spaced between −2 and 2, and the histogram is Gaussian smoothed with an s.d. of four bins. These choices were found to result in sufficient accuracy in estimating the trough of the distribution for all of the units. To score the degree of bimodality, we find three important values in the histogram: the peak of the negative portion, the trough around 0 and the peak of the positive portion. First we find the trough *x*_min_ at position *i*_min_ in the bin range of 175 to 225 (corresponding to the center bins for the 400-bin histogram). Then we find the peaks *x*_1_, *x*_2_ in the bin ranges from 0 to *i*_min_ and from *i*_min_ to 400. The bimodality score is defined by

$${{{\rm{bimod}}}}=1-\max ({x}_{\mathrm{min}}/{x}_{1},{x}_{\mathrm{min}}/{x}_{2})$$

In other words, we compare the density of the *x*_proj_ distribution at its trough to the peak densities for both clusters. If the density at the trough is similar in value to the density of either the left or right peak, this indicates a nonbimodal distribution.

#### Refractory auto- and cross-correlograms

There are many cases where the regression axis has a bimodal distribution, yet the clusters are part of the same neuron. This is due to the nonstationarity of the waveforms from the same neuron, either due to drift or due to other factors. In such cases, we need to use extra information such as the statistics of the spike trains. Fortunately, all neurons have a refractory period, which is a short duration (1–5 ms) after they fire an action potential when they cannot fire again. The refractory period is heavily used by human curators to decide whether (1) a cluster is well isolated and not contaminated with spikes from other neurons; and (2) a pair of clusters are distinct neurons or pieces of the same neuron. These two decisions can be made based on the auto-correlograms (ACGs) and CCGs, respectively:

$$\begin{array}{rc}{{{\rm{ACG}}}}(\delta t)&=\displaystyle\mathop{\sum}\limits_{k,j,{s}_{k}-{s}_{j}=\delta t}1\\ {{{\rm{CCG}}}}(\delta t)&=\displaystyle\mathop{\sum}\limits_{k,j,{s}_{k}-{r}_{j}=\delta t}1\end{array}$$

where *s*_*k*_, *r*_*j*_ represent the spikes times of the two neurons. In practice, we bin the ACGs and CCGs in 1-ms bins from *δt* = −0.5 s to *δt* = 0.5 s. We consider the central bins of the CCGs and calculate how likely it is to see a very small number of coincidences in that bin if the two clusters are from neurons firing independently from each other. We define *n*_*k*_ as the number of coincidences in the central −*k* to +*k* bin range, *R* as the baseline rate of coincidences calculated from the other bins of the CCG. CCGs may be asymmetric and to account for that we estimate *R* as the maximum rate from either the left or right shoulder of the CCG. We use two criteria to determine refractoriness. The first criterion is simply based on the ratio of refractory coincidences versus coincidences in other bins, which works well in most cases, except when one of the units has very few spikes, in which case very few refractory coincidences may be observed just by chance. For the first criterion, we use the ratio *R*_12_ of *n*_*k*_ to its expected value from a rate *R*, where *R*_12_ takes the minimum value of this ratio across *k*. We set a threshold of 0.25 on *R*_12_ to consider a CCG as refractory and 0.1 to consider an ACG as refractory. For the second criterion, we use the probability *P*_*k*_ that *n*_*k*_ spikes or less would be observed from a Poisson process with rate *λ*_*k*_ = (2*k* + 1)*R*, which we approximate using a Gaussian with the same mean and s.d. as the Poisson process as

$${P}_{k}=\frac{1}{2}\left(1+{{{\rm{erf}}}}\left(\frac{{n}_{k}-{\lambda }_{k}}{{\left(\epsilon +2{\lambda }_{k}\right)}^{1/2}}\right)\right).$$

where *ϵ* = 10^−10^ is a small constant to prevent taking the square root of 0. If ${Q}_{12}=\min ({p}_{k})$ is large, it implies that the number of refractory spikes have a high chance of being observed from a Poisson distribution with the baseline rate and thus the CCG is not refractory. We set a threshold on *Q*_12_ of 0.05 to consider a CCG as refractory and 0.2 to consider an ACG as refractory. Both criteria have to be satisfied for a CCG to be refractory: *R*_12_ < 0.25 and *Q*_12_ < 0.05 for the CCG and *R*_12_ < 0.1 and *Q*_12_ < 0.2 for the ACG. The different thresholds for ACG and CCG have to do with the function of these decisions: for the ACG, we want small contamination rates *R*_12_ because this indicates a well-isolated neuron, whereas for the CCG we want to prevent clusters from being split if their contamination rate *R*_12_ is indicative of a relationship between these two clusters. This is similar for *Q*_12_.

#### Global merges

Global merges are performed after all sections of the probe have been clustered. As a similarity metric, we use the maximum correlation of pairs of waveforms over all timelags. To test for merges, we sort all units by their number of spikes and start testing in order from the units with the most spikes. For each unit, we find all other units with a similarity above 0.5 and start testing for merges starting from high to low similarity. A merge is performed if the CCG is refractory. After a merge is performed, the merged unit is retested versus all other units with a similarity above 0.5. After no more merges can be performed, a unit is considered ‘complete’ and is removed from potential merges with subsequent tested units.

### Scaling up the graph-based clustering

Graph-based clustering algorithms do not scale well with the number of data points and we had to develop new formulations and optimization strategies. The poor scalability is due to several problems: (1) finding the neighbors of all points scales quadratically with the number of points; (2) the *k-*nearest neighbors in a small dataset are relatively further away from the *k-*nearest neighbors in a larger dataset; and (3) existing optimization algorithms like Leiden/Louvain are inherently sequential and thus hard or impossible to parallelize on GPUs. The first problem could be reduced by using some of the neighbor-finding algorithms that have sublinear time for finding neighbors^30^; however, for the particular type of data that we consider, we find these algorithms to be slower, not faster, than the brute force approach, at least when a multi-core CPU is used. The second problem is an issue because the effective neighborhood size around a point influences its clustering properties. If the neighborhood sizes are very small, clusters may split up into multiple pieces more easily. If it is too large, it may include points from other clusters. As a recording grows in duration, the number of spikes grows linearly with it. Thus, some normalization step must be introduced to ensure that neighborhood sizes are comparable for short and long recordings. To solve the third problem, a redesign of the cost function is necessary, so as to make multiple optimization steps in parallel.

Our approach for improving scalability relies on a subsampled data approach, where we only search for neighbors in a smaller subset of all points. In other words, instead of constructing an *N* × *N* adjacency matrix, where *N* is the number of points, we construct an *N* × *n*_sub_ adjacency matrix, where *n*_sub_ is a fixed number of spikes independent of recording length, which is determined by the size of the section of the probe being clustered (40 μm typically, for which we use *n*_sub_ = 25,000). This solves the first two problems, but not the third. To solve the third problem, we replace the standard adjacency graph with a bipartite graph, which includes ‘left’ nodes and ‘right’ nodes. All connections are between a left node and a right node. The left nodes are defined simply as all points in the data. The right nodes are a copy of the subsampled nodes and their edges to the left nodes are defined by the adjacency structure of the original subsampled nodes. Edges thus only exist between original nodes and copies of the subsampled nodes, thus making the graph bipartite. The reason for making the graph bipartite is to allow the cluster identities for left nodes to be optimized independently of each other, given the identities of the right nodes, and vice versa. The modularity cost function must also be slightly modified for the bipartite graph from:

$${{{\mathcal{H}}}}=\displaystyle\frac{1}{2m}\mathop{\sum}\limits_{ {c}}\left({e}_{{c}}-\gamma \frac{{({K}_{c}^{\mathrm {left}}+{K}_{c}^{\mathrm {right}})}^{2}}{2m}\right)$$

into:

$${{{\mathcal{H}}}}=\displaystyle\frac{1}{2m}\mathop{\sum}\limits_{c}\left({e}_{c}-\gamma \frac{{K}_{c}^{\mathrm {left}}{K}_{c}^{\mathrm {right}}}{2m}\right)$$

where ${K}_{ {c}}^{\mathrm {left}}$ is the sum of degrees of left nodes in the cluster *c*, ${K}_{{c}}^{\rm{right}}$ is the sum of degrees of right nodes and *e*_*c*_ is the number of edges between left and right nodes. If the cluster identities for all right nodes are fixed, a short calculation shows that every left node *t* can be assigned independently to a cluster *σ*_*t*_ to maximize their contribution to the modularity cost function:

$${\sigma }_{t}={{{{\rm{argmax}}}}}_{j}\left({n}_{tc}-\gamma \frac{{k}_{t}{K}_{c}^{\mathrm {right}}}{2m}\right)$$

where *n*_*tc*_ is the number of right node neighbors of left node *t* in cluster *c* and *k*_*t*_ is the degree of node *t* like before. Similarly, every right node can be assigned independently given fixed assignments for all left nodes. Thus, we can iterate between assigning cluster identities to all right nodes given all the left nodes, followed by assigning all the left nodes given all the right nodes. Note that a left node which represents the same point as a right node may in fact be assigned to a different cluster than its corresponding right node. This new iterative optimization has massive parallelism and thus is suitable for GPU acceleration.

This optimization is initialized with 200 clusters identified by *k*-means++, which we implemented in pytorch for GPU-based scalability^44^.

### Benchmarking

The benchmarking procedures and algorithm parameters were the same for the hybrid simulation, biophysical simulation and drift simulation.

#### Performance metrics

Each ground-truth unit was compared to the 40 closest detected units from the algorithm, where closeness was defined by the distance between the ground truth and detected units’ best channels. If an estimated spike from a detected unit was less than or equal to 0.2 ms from a ground-truth spike it was counted as a positive match. The FP rate was defined as the number of estimated spikes without a positive match divided by the total number of estimated spikes. The FN rate was defined as the number of missed ground-truth spikes divided by the total number of ground-truth spikes. We matched the ground-truth unit with the detected unit that maximized the score, defined as 1 − FP − FN (ref. ^6^). The upper bound of the score is 1. In Fig. 4e–j, the ground-truth units were sorted by their score from each algorithm separately. We defined ground-truth units as being correctly identified in Fig. 4j if the score was higher than 0.8.

To determine the rate of false positive units returned by the spike sorters, we used a classification criterion based on the ACGs. Using the same ACG metrics as above, we classified units as ‘good’ if their estimated refractory violations had a rate < 0.2. This is also the default rate in Kilosort4 to call units ‘good’ and we used the same strategy for labeling units from the other algorithms. Focusing only on good units had a negligible impact on the number of matches between the algorithm and the ground truth. In other words, if a unit matches the ground truth well, it is also very likely that it has a refractory ACG, because the ground-truth units have refractory ACGs. Units which were classified as ‘good’ by each algorithm and did not match any ground-truth units were instead determined to be false positive units (not to be confused with the false positive rate of spikes in the previous paragraph).

#### Spike-sorting algorithm parameters

We ran Kilosort1, 2, 2.5 and 3, IronClust, MountainSort4, SpyKING CIRCUS, SpyKING CIRCUS 2, HDSort and Herding Spikes on all simulations using the SpikeInterface platform to ensure that all spike-sorting algorithms were run in the same way. For Kilosort1, 2, 2.5 and 3, we set the detection thresholds to [9, 8] instead of their defaults, which varied across versions. Also, to speed up Kilosort1, we set the number of passes through the data to two instead of six (this did not reduce performance).

For the other top-performing algorithms (IronClust, MountainSort4 and SpyKING CIRCUS), we ran a parameter sweep over the detection threshold and used the detection threshold which maximized the number of correctly identified units on the medium drift simulation. For MountainSort4 and IronClust, the best detection threshold was the default detection threshold; for SpyKING CIRCUS, this was a detection threshold of 4.5. For SpyKING CIRCUS 2, we noticed poor detection of low norm units (Extended Data Fig. 7b,e) and thus also swept over the detection threshold for this algorithm, but did not achieve an improvement in performance. For IronClust the default adjacency radius is 50, whereas for MountainSort4 the default is set to all channels. This large radius led to an incredibly long runtime (tens of hours) and thus we set the MountainSort4 adjacency radius to 50 as well.

All other parameters were set to their default values.

### Hybrid simulation

We also created ‘hybrid ground-truth datasets’. These datasets are created using ground-truth units or manually curated units^6,14,45^. These units can be inserted into other real recordings or the same recording in a different position to ensure appropriate background noise. Multiple ground-truth units can be inserted in a dataset in this fashion; however, if the dataset drifts, then the waveforms must also be inserted with drift in some way; otherwise, the simulation is inconsistent. Instead, we chose to use recordings with low drift to avoid these issues, choosing three such recordings from the IBL dataset, each from different brain areas (Fig. 3a)^22^.

In brief, for each recording, we ran Kilosort4 with default parameters to get spike times for extracting waveforms, and then re-inserted these waveforms into the same recording at different positions. We only used waveforms from units with a contamination ratio less than 0.1. We used the estimated spike times for these units to compute the average waveforms in the raw recording. One hundred of these waveforms were randomly chosen to be added to the real recording, at either eight sites above or eight sites below the original position. We simulated the spike trains with an exponential random inter-spike interval (ISI) and an average ISI of ~500 ms. The ISI increased and decreased throughout the recording to match the firing rate fluctuations of the recording at the position on the probe on which the simulated unit was placed. The firing rate of the probe was computed in 100-ms bins for each channel, where each detected unit was assigned to its biggest channel. The firing rate was then smoothed across channels with a Gaussian filter with an s.d. of ten channels and set to mean of 1 for each channel, and then the ISI was multiplied by the inverse of this value, with a minimum ISI of 2 ms as the refractory period. The ISI defined the simulated spike times and the waveform for the unit was then added at each of the spike times to the original recording (Fig. 3b). In Fig. 3, for visualization purposes, we show the spikes and the background high-pass filtered with a 300-Hz cutoff and whitened with the whitening matrix from the real recording.

We ran the ten different spike-sorting algorithms on the three different hybrid ground-truth simulations and combined the results across the three simulations in Fig. 3c.

### Biophysical simulation

We investigated the biophysical simulation from the SpikeInterface paper^25^, available at DANDI 00028 (Extended Data Fig. 3b). We first quantified the waveforms from a real recording (ID 3f6e25ae-c007-4dc3-aa77-450fd5705046) and from the biophysical simulation. In the real recording, we used the spike clusters extracted using Kilosort4 with good refractory periods (contamination ratio <0.2). We used the spike times from these clusters and computed the mean waveform shape across all spike times on the recording high-pass filtered in time at a cutoff frequency of 100 Hz. For the biophysical simulation, we used the ground-truth spike times to compute the mean waveform shape on the simulation, also high-pass filtered in time at a cutoff frequency of 100 Hz (Extended Data Fig. 3c,d).

We computed the trough-to-peak time (T2P) for each waveform using the channel with the largest norm (the best channel) and finding the time between the minimum waveform value and the first time after the minimum in which the waveform decreased (Extended Data Fig. 3e). We computed the spatial spread of each waveform by determining the channel with the largest distance from the best channel which has a minimum value that is less than half the minimum from the best channel (Extended Data Fig. 3e).

We ran Kilosort2 on the biophysical simulation at normal speed, as carried out in previous work^25^, and also ran it on the biophysical simulation at twice the normal speed. We sped up the simulation by subsampling, taking every second sample. We set the sampling frequency in Kilosort2 to 32,000 in both cases. For the sped-up simulation, we set the time bin for estimating the contamination ratio to 0.5 ms instead of 1 ms. We then benchmarked the quality of the Kilosort2 using the ground-truth spike times in the simulation in both cases (Extended Data Fig. 3g). Detected units were defined as having good refractory periods if their contamination ratios were less than 0.2 (Extended Data Fig. 3h). False positives were detected units with good refractory periods which did not match any ground-truth units (Extended Data Fig. 3f,h).

### Drift simulation

To determine the performance of various spike-sorting algorithms, we created realistic simulations with drift using the properties of 512 electrophysiological recordings from the IBL performed using Neuropixels1.0 probes^15,22^. These recordings were processed by the IBL using pyKilosort. The simulation generation was over two times faster than real time (for example a 45-min simulation took around 20 min to generate), which enabled us to create several simulations for benchmarking. The simulations, other than ‘step drift, aligned’, used the site configuration of the Neuropixels1.0 probes, which have a vertical spacing of 20 μm, a horizontal spacing of 32 μm and a horizontal offset across rows of 16 μm.

pyKilosort, like other Kilosort versions, returns the estimated depth for each processing batch at nine equally spaced positions along the 3.84-mm probe. The processing batch size for all IBL recordings was 65,536 time points. We quantified the drift range for each recording by first taking the median of the depth across the nine positions, then computing the difference between the fifth and 95th percentile of the drift. We used the properties of the drift across these recordings to create simulated drift (see drift examples in Extended Data Fig. 5).

For the simulations, we generated a drift trace of length 45 min at each of the positions, then upsampled the drift to all 384 channels using linear interpolation. The drift was the same across a period of 2 s for all simulations, other than the fast drift simulation which varied in periods of 200 ms. Here are the details of the generation of each drift simulation:

- No drift: zero drift at all nine positions.
- Medium drift: the overall drift was generated as random Gaussian noise smoothed in time with a Gaussian filter of *σ* = 100 s. Drift at each of the nine positions was generated as random Gaussian noise smoothed in time with a Gaussian filter of s.d. of 100 s and smoothed across the positions with a Gaussian filter with *σ* = 2. This per-position drift was rescaled by a factor of 0.4 and added to the overall drift, then the drift across positions and time was rescaled such that the minimum and maximum values were −7 μm and 7 μm. This resulted in a simulation with a drift range of 9.4 μm.
- High drift: the overall drift and per-position drift were generated in the same way as the medium drift. The per-position drift was next rescaled by a factor of 0.26 and added to the overall drift, then the drift across positions and time was rescaled such that the minimum and maximum values were −18.5 μm and 18.5 μm. This resulted in a simulation with a drift range of 27.9 μm.
- Fast drift: a medium drift simulation was used for the slow drift across positions and time (generated in bins of 2 s, then upsampled to 200-ms bins with nearest neighbor interpolation). Then fast drift events were generated with an amplitude of 10 μm and a difference of exponentials kernel with a rise time of 80 ms and a decay time of 200 ms. Then, 300 of these fast drift events were added to the upsampled medium drift simulation at random times.
- Step drift: the overall drift and per-position drift were generated in the same way as the medium drift. The per-position drift was next rescaled by a factor of 0.58 and added to the overall drift, then the drift across positions and time was rescaled such that the minimum and maximum values were −4 μm and 4 μm. Halfway through the recording, 30 μm was added to all the drifts across positions.
- Step drift, aligned: same exact drift as step drift, but the waveforms were upsampled using aligned probe sites with a vertical separation of 20 μm and a horizontal separation of 32 μm.

#### Extraction of waveforms at multiple depths

Obtaining waveforms across many depths requires recordings with substantial drift. In the IBL dataset we found 11 such recordings with high drift that sampled a range of 40 μm in depth. We preprocessed the recordings by whitening and high-pass filtering with a cutoff of 300 Hz. We then used the estimated spike times from pyKilosort for each detected unit in these recordings and the estimated depth of the probe to compute the average waveform for the unit at specified depth positions. We used 20 depth bins each of size 2 μm, resulting in average waveforms across 40 μm. To ensure the quality of the waveforms, we did not use any units that had fewer than 50 spikes at each depth.

The waveforms were denoised by reconstructing each waveform across depths with only its top three PCs. The waveforms were then normalized by the average norm of the waveform across depths. We then threw out waveforms that varied substantially from −20 μm to 20 μm in depth, as these waveform shape changes are likely caused by other processes besides drift. To quantify the variation across depth we computed the Euclidean distance across channels and time points between the waveform at −20 μm and the waveform at 20 μm shifted up by four channels (a distance of 40 μm). We removed units with variation greater than 0.25 (~25% of units), resulting in a waveform bank of 597 units from the 11 recordings.

Next we needed the waveform shapes at a finer scale than 2 μm. For this, we upsampled the waveforms by a factor of 100 using kriging interpolation^13^ with a regularization coefficient of 0.01 and a Gaussian s.d. of 20 μm. For the step drift with aligned site simulation, the upsampled waveforms were interpolated using a probe with sites aligned vertically. Then the waveforms were again normalized by the average norm of the waveform across depths. We next divided these waveforms into two groups according to the contamination rates from their units’ estimated spike trains^10^: a contamination rate less than 0.1 were used to generate ‘single-units’, whereas those with a contamination rate greater than 0.1 were used to generate ‘multi-units’.

The units from these recordings exhibited waveform changes across depth (see example waveforms in Fig. 4c and Extended Data Fig. 4a). All waveforms moved down the probe as the depth changes, but some waveforms also changed their shape (example units 2 and 4, which had smaller spatial footprints). This shape change could not be inferred by other channels. We demonstrated this by using the same kriging interpolation procedure as above to estimate the 0-μm depth waveform from the waveforms at other depths (Extended Data Fig. 4b). The waveform at 0-μm depth was well-estimated for units 1 and 3 but not for 2 and 4. This exemplifies the need for real recordings to create accurate simulations of waveform shapes.

We quantified the performance of the spike-sorting algorithms as a function of the spatial extent of the waveforms of the ground-truth unit (Extended Data Fig. 7c,f). We defined the spatial extent of the waveform as the spatial scale across channels over which the waveform shape is maintained (using the 0-μm depth waveform). To compute this, we first matched the waveform with its most similar waveform from the simple templates as defined by cosine similarity. We then projected the waveform onto this best-template waveform and thresholded it to obtain a template weight for each channel. We next computed the weighted mean of the distance from each channel to the center of mass of the waveform, as defined by the template weights, and termed this the spatial extent.

#### Simulation of spikes

We simulated 600 ‘single-units’ and 600 ‘multi-units’ by randomly drawing waveforms from these two classes. These waveforms were randomly placed on the probe at positions from site 4 to site 380. To create the correct waveform shapes, the waveform’s best channel modulo 4 was computed and maintained in the simulation (because the probe site arrangement repeats every four sites).

We used the ISIs from detected units in the 11 recordings that had a contamination rate of less than 0.1; this was 1,497 units in total. The average firing rate of these units was 12.6 Hz. Each simulated spike train for a ‘single-unit’ was then generated by randomly shuffling the ISIs of one of the detected units. For the spike trains of ‘multi-units’ we generated Poisson spike trains with firing rates drawn randomly from these units’ firing rates.

The norms for the ‘single-units’ were generated by adding a constant (10) to a random exponential with a mean of 7, which approximated the distribution from units detected in the data. The norms for the ‘multi-units’ were generated from a uniform random distribution with a range from 4 to 10. The waveform across depth for each unit was then multiplied by its norm. We quantified the performance of the spike-sorting algorithms as a function of the norm of the ground-truth unit (Extended Data Fig. 7b,e).

We then added the spike train of each simulated unit one by one to the simulation using the simulated drift at each time point to determine which depth of the waveform to add for each spike. Collisions could occur in the spike trains, so we added the spike train in three interleaved parts to ensure correct reconstruction, while still maintaining the speed of simulation generation.

All simulations used different waveforms, spike trains and norms, except for the two-step drift simulations, in which all parameters were kept fixed to determine the effect of probe site configuration. These two-step drift simulations therefore only differed in their exact waveform shapes across depths due to the difference in the probe site positions.

#### Simulation noise and ‘unwhitening’

We added random noise, with a flat frequency spectrum in time up to 300 Hz, to each channel in the simulation. This noise was scaled to have an s.d. of 0.76. Next the simulation was ‘unwhitened’: the simulation was multiplied by the inverse of a whitening matrix estimated from one of the 11 recordings used. Different whitening matrices were used for each simulation, except for the two-step drift simulations, where it was the same matrix for both. Finally, to save the simulation as int16, the simulation was multiplied by 200, cut off at ±32,767 and converted to int16. For each simulation we saved a corresponding ‘.meta’ file, which SpikeInterface expects for processing IMEC Neuopixels probe recordings. For the aligned site probe, we added a probe type to the spike GLX loader in SpikeInterface. The unwhitened simulation is shown in Extended Data Fig. 4a in comparison to a real recording high-pass filtered in Extended Data Fig. 4b (we cannot ‘un-high-pass’ filter the simulation).

#### Comparison to other benchmarking approaches

Here we compare our approach to previous spike-sorting benchmarking performed in the literature. The first approach is to use datasets where the ground-truth spiking of a single unit is known. These datasets are acquired by performing cell-attached recordings while simultaneously recording with a probe. Then spike sorting is performed on the probe and compared to the ground-truth spiking to determine spike-sorting performance. As these are very difficult experiments, existing ground-truth datasets were acquired in anesthetized animals and are very short^2,46–52^. This makes these datasets much easier to spike sort compared to long, realistic awake recordings with drift and with relatively more neuronal firing. When SpikeForest used these ground-truth datasets to compare various spike-sorting algorithms (‘PAIRED’ recordings, https://spikeforest.flatironinstitute.org/)^45^, they found that IronClust, Kilosort2 and SpyKING CIRCUS performed similarly on these recordings. This is consistent with our own benchmarking results on the ‘no drift’ recordings, where many of the spike-sorting algorithms recovered units with high norms equally well (Extended Data Fig. 7b,e); however, most recordings in awake animals have drift and contain many low norm units that can be isolated by Kilosort.

Another approach is to create so-called ‘hybrid ground-truth datasets’. Either ground-truth units, as acquired above, or manually curated units are used^6,14,45^. These units can be inserted into other real recordings, or the same recording in a different position after being subtracted off, to ensure appropriate background noise. Multiple ground-truth units can be inserted in a dataset in this fashion; however, these hybrid datasets depend on finding the neurons in the first place and they also depend on correcting for the initial drift of the dataset. Alternatively, these ground-truth units can be used to create simulations with drift. Such simulations must account for two important properties: (1) waveform shapes change as the electrode moves (as demonstrated in Extended Data Fig. 4a,b) and (2) the background noise must ‘look’ like background neurons. To accomplish (1), we obtained waveforms at various drift positions from real recordings, as outlined above, to simulate the waveforms at various depth positions. To accomplish (2), we added 600 ‘multi-units’ with low norms to the simulation to create more realistic background, on top of adding Gaussian noise with a matched frequency spectrum (Extended Data Fig. 4c).

The final approach is to instead simulate waveforms, either using some specified properties^3^ or using the electrical field of a biophysically simulated neuron^53–56^. These simulators do not produce waveforms as diverse as real neurons from recordings, likely because we lack a full understanding of how the tissue geometry interacts with action potentials and the probe to create all the diverse spike shapes that can be observed. Various types of noise and background can be added to these neurons. For example, these simulated neurons can be added to background signal from other recordings^3^. Alternatively, noise can be added by simulating neurons further away from the probe^55^. Other simulators use spatially correlated noise with parameters extracted from the data^53,56^. The MEAREC simulator includes the option for probe drift; however, it is unclear how much the waveform shape changes over drift positions in their simulations as this depends on the geometry of the electrical fields.

### Other probes

We illustrated Kilosort4 results on two other types of recording devices. First, we used the DANDI dataset 000231 (https://dandiarchive.org/dandiset/000231/0.220904.1554), which contains data from previous work^26^, recorded with 64-channel linear silicon probes (Cambridge Neurotech H3). These probes have contacts arranged in a single column with 20-μm vertical spacing and an 11 × 15 μm contact area. The probe spanned layers 2/3 to layer 6 of mouse barrel cortex in a headfix preparation. Second, we used the DANDI dataset 000410 (https://dandiarchive.org/dandiset/000410/draft) from previous work^27^, which was recorded with 32 independent tetrodes driven by an implanted microdrive targeting area CA1 of the dorsal hippocampus in freely moving rats.

### Reporting summary

Further information on research design is available in the Nature Portfolio Reporting Summary linked to this article.

## Online content

Any methods, additional references, Nature Portfolio reporting summaries, source data, extended data, supplementary information, acknowledgements, peer review information; details of author contributions and competing interests; and statements of data and code availability are available at 10.1038/s41592-024-02232-7.

### Supplementary information

Supplementary Information Supplementary Note.

Reporting Summary

## Data Availability

We used datasets shared by N. Steinmetz and the IBL^16,22^ (available at https://rdr.ucl.ac.uk/articles/dataset/Recording_with_a_Neuropixels_probe/25232962/1 and https://ibl.flatironinstitute.org/public/). We also used datasets from the DANDI archive at https://dandiarchive.org/dandiset/000410/draft (ref. ^26^) and https://dandiarchive.org/dandiset/000231/0.220904.1554 (ref. ^27^). The simulated datasets are shared at 10.25378/janelia.25298815.v1.

## Code availability

Kilosort4 is available at 10.5281/zenodo.10863771 under a GPL-3 license^57^ (v.2, 2.5 and 3 will remain available at the same link as downloads under GPL-2 licence).
