# Supplementary material for: Spike sorting with Kilosort4
Source: Nat Methods. 2024 Apr 8;21(5):914–21. doi: 10.1038/s41592-024-02232-7 (PMC11093732; doi:10.1038/s41592-024-02232-7)
Supplement: Supplementary file 1 — Supplementary Note. [file 41592_2024_2232_MOESM1_ESM.pdf]

nature methods

Article

https://doi.org/10.1038/s41592-024-02232-7

Spike sorting with Kilosort4

In the format provided by the authors and unedited

Algorithms for Kilosort 2/2.5/3

This supplementary information describes algorithms from previous versions of Kilosort which are no longer used in Kilosort4, but which have not been previously described. These are divided into drift tracking (Kilosort2), global optimization (Kilosort2/2.5) and recursive pursuit (Kilosort3).

Drift tracking (Kilosort 2)

Drift tracking was an alternative strategy of accounting for drift. Unlike the drift correction algorithms from Kilosort2.5 and onwards, drift tracking does not require a geometrical model of the recording channels and thus it can be used for recordings with tetrodes, single electrodes etc. Drift tracking works well when drift changes are continuous, or at least the drift positions overall span a continuous range. Drift tracking does not work well when the recording consists mainly of two drift positions, with little sampling of the positions in-between (see step drift benchmarks in Figure 4). Drift tracking requires two algorithmic steps, described below: online template learning and fast drift tracking.

Online template learning and tracking

In the simplest case, imagine that the drift of the probe is very slow. A possible spike sorting strategy in that case could be to start by spike sorting a subsection of the data, say 5 minutes, over which the probe is at an almost fixed position, since drift is slow. With the templates learned from 5 minutes of data, one could then use the templates to extract and assign spikes on the next 5 minutes of data, and update the templates based on the spikes that were found. If the drift is slow, the distribution of waveforms from single templates will have only shifted slightly, so that spike assignments to clusters are still correct. The update of the templates would then track the mean of the shifted distribution for each cluster. This is, in a broad sense, the drift tracking strategy from Kilosort2, and it was a natural extension of the online template learning of Kilosort1. In the rest of this section, we describe the exact mathematical form of online template learning.

The generative model of Kilosort is given by the reconstruction cost function:

$$\text{cost}(\mathbf{W},\sigma,\mathbf{s},\mathbf{x}) = \sum_{ct}\left(V(c,t) - \sum_k x(k)\cdot W_{\sigma(k)}\left(c,t-s(k)\right)\right)^2$$

where $V(c,t)$ is the recorded voltage at channel c and timepoint t, $\sigma(k)$ is the template index for spike k at time $s(k)$, W_i is the multi-channel template for cluster i and $x(k)$ is the norm of spike k. For mathematical simplicity, we assume "infinite" temporal windows for each template W_i and we also assume that they span all channels of the probe, but in practice we restrict each template to a width of $n_t = 61$ samples and to a small number of channels (typically 32). Learning and inference in this model proceeds via the standard "EM-style" algorithm. For inference, we assume the templates W are fixed, and we simultaneously infer $s(k),\sigma(k),x(k)$ for all k via the parallelized matching pursuit algorithm described above. Learning proceeds iteratively by inferring $\mathbf{s},\sigma,\mathbf{x}$ from a single batch with the current W, and computing an improved W for this batch:

$$W_i^{\text{batch}}(c,t) = \sum_{k,\sigma(k)=i} V(c,s(k)+t)/n_i$$
$$n_i = \sum_{k,\sigma(k)=i} 1$$

where we omit the dependence on norms $x(k)$ for robustness, since outlier artifacts may have very large x_k that could dominate the templates. To convert this EM-style algorithm into an online algorithm, we perform the inference at a single batch level ($\approx$ 2 sec), and update the templates with an exponential filter which depends on the number of spikes inferred for each template:

$$W_i^{new} = p_i W_i^{old} + (1-p_i)W_i^{\text{batch}}$$
$$p_i = \exp(-n_i/\tau)$$

where τ is typically set to 400 spikes. In other words, it takes approximately 400 new inferred spikes for W_i to "forget" its previous value. For learning to be effective, the batches from one recording are processed in pseudorandom order in Kilosort1. For tracking in Kilosort2, we fix the order of the batches. For example, if the batches were processed in consecutive order 1, 2, 3, etc, then online learning of the templates would ensure tracking of the slow changes in templates over long timescales, similar to the simple scenario describe at the beginning of this section. In practice however, drift often contains fast components in addition to slow components. Since the τ scale is set to 400 spikes, fast drift cannot be tracked well with this approach. Reducing τ would improve tracking, at least for neurons with high firing rates, but many neurons fire at $\sim$ 1Hz and/or in bursty sequeces. For such neurons, drift movements <1 min would be quite difficult to track, since very few spike samples of the neurons are seen in that time.

Tracking fast drift

To track fast drift, we make another modification to the online template learning algorithm. Instead of processing the batches in random order (like in Kilosort1), or in consecutive order (like for slow drift), we use a special re-ordering of the batches which puts similar batches next to each other. We define and estimate a drift dissimilarity metric between batches, based on the distributions of spike shapes in each batch.

To construct the drift similarity metric, the first step is to extract a set of templates $\mathbf{W}^k$ for each batch k. These templates are obtained by first detecting spikes via threshold crossing of PC-projected data. The PC projection is performed by convolving the data with the top three PCs, squaring and adding together the projections. Local maxima in this projection are found in a neighborhood of the nearest 17 channels and 61 timepoints. The features are extracted at the local maxima via PC projection for a subset of neighboring channels. Scaled k-means clustering is then performed, which is initialized with a random subset of the spikes and implemented on the GPU for speed, since it needs to be performed once for each 2 sec batch. A fixed number of clusters is used, equal to half the total number of channels. The centroids of the clustering are used as templates.

Once the templates $\mathbf{W}^k$ are obtained for each batch, we calculate a dissimilarity matrix A_{ij} between each pair of batches, each with their own template sets W^i, W^j:

$$A_{ij} = \sum_k \text{min}_l \|W_k^i - W_l^j\|^2$$

In other words, A_{ij} is a metric which is small when every template k in a set of templates W^i has a close match in another set of templates W^j. Pairs of batches i, j from similar drift levels will therefore have a small dissimilarity, while batches taken at very drift levels will have high dissimilarity because the templates won't be very well matched.

Once we compute the matrix $\mathbf{A}$, all that is left is to find a permutation ρ of the batches in which small dissimilarity values $A_{\rho(i)\rho(j)}$ are near the diagonal. The algorithm we use for this is a version of "rastermap", a framework algorithm we have been developing for sorting high-dimensional data along a one-dimensional continuum. This particular version of rastermap matches the similarity matrix $\mathbf{A}$ to the matrix of distances in a one-dimensional space, where x_k is a scalar value assigned to each batch k and optimized by the algorithm:

$$\hat{x} = \text{argmin}_x \sum_{ij} (A_{ij} - d'_{ij})^2$$

$$d_{ij} = -\log(1 + (x_i - x_j)^2)$$

$$d'_{ij} = d_{ij} - < d_{ij} >_{ij}$$

where the $< \cdot >$ operation signifies averaging. This cost function is initialized with x_k based on the largest left singular vectors of $\mathbf{A}$ and minimized by gradient descent. We preprocess $\mathbf{A}$ by z-scoring each row separately and symmetrize it by adding its transpose to it. In the optimization we ignore the mean of d_{ij} over all i, j, to avoid having to fit a constant offset term. Once the gradient descent optimization converges, we obtain a sorting of the batches by $\rho = \text{argsort}(\mathbf{x})$, where the argsort operation returns the index order of a vector.

This ordering ρ is used to perform online template learning and tracking. The template learning is performed by running the algorithm over one half of the data (typically the first half after reordering, from the middle of the ρ range to the first batch and then back to the middle of the range). During this stage, the templates are being learnt: new templates can be introduced from the residuals of the reconstruction process, templates which are not used above a baseline spike rate are discarded, and merges and splits are also performed. See the next section for this template learning step. Once the template set is learned, the tracking is performed from the middle of the ρ range to the first batch, and then from the middle of the ρ range to the last batch. During the tracking phase, no new templates can be created or destroyed with any of the operations performed in the template learning phase. However, the template waveform itself continues to change in an online fashion as described above in the online template learning section.

Global optimization algorithms for clustering (Kilosort 2 / 2.5)

Drift tracking was not the only new algorithm introduced in Kilosort2. We also added algorithmic steps designed to perform global optimization moves, in order to escape local minima which are very common in clustering algorithms. These global optimization moves were of three types: initialization, splitting a cluster and merging two clusters. We describe these in separate sections below. Some global optimization moves were also performed in Kilosort1 (simple splits and merges), but they were not as important there because the automated results of Kilosort1 underwent manual curation in Phy, and thus an oversplit cluster distribution was preferred because merges are

much easier than splits. For Kilosort2 however, the automated results of the algorithm became sufficiently good to be used in an automated manner, and thus it was important to avoid oversplit or overmerged clusters.

Template initialization from residual

Initialization is one of the most important steps in a clustering algorithm. A common initialization for k-means style algorithms is k-means++, which sequentially adds data points as cluster centroids if they are far enough away from centroids already chosen. We use a similar strategy in Kilosort2, with the added complication that spikes which are far from existing centroids would not even be detected by the online template matching step. In this case, such spikes must be detected in the residual of the model after reconstructing the data with the spikes found by template matching.

To perform these detections, we run a spike detector on the residual and pick a subset of those spikes as new templates to be introduced in the optimization. The spike detector was designed primarily to be fast, and to ensure that large norm spikes are not missed. It uses six single-channel prototype waveforms $w_k, k = 1, 2, ..., 6$. For each channel, we check the variance explained of all templates that extend over the nearest n channels with $n \leq 7$ and have the same single-channel waveform on each channel, chosen from one of the six single-channel prototypes w_k. This is a much simplified version of the spike detector introduced in Kilosort2.5, but a very fast version nonetheless. Similar to the Kilosort2.5 spike detector, this detector computes the maximum variance explained at each channel and each timepoint, that can be obtained using one of the 42 template combinations described above. Using this maximum variance matrix, we find peaks that are maxima across channels for each timepoint, and then we find the subset of those which are also maxima across time, and in a neighborhood of ± 4 channels in a single batch. The reason for taking the maxima across time is to ensure that no spike from the same neuron is detected twice, because these spike detections are introduced as new putative templates.

New templates are introduced on every batch. The raw data snippets at the detected spikes are first smoothed with three principal components before being added to the set of active templates. The number of spikes detected by each template is monitored using an exponential filter with a decay scale of ~ 20 batches. Every five batches, templates are triaged and removed from the active set if their firing rate is below 0.02 spikes/s. During this step, templates are also merged together if they have a high correlation ($>$0.9) and if their means are similar ($< 4\sqrt{10}$ difference). If a merge is performed. the template with the smaller firing rate is simply dropped out of the active set.

Bimodality splits

Merges are relatively easy to perform, for example by checking all pairs of correlated templates and computing their cross-correlograms to find whether it is refractory (as described above for Kilosort4). Splits however are much more difficult, because finding a good split of a cluster in high-dimensional space is in itself a combinatorially difficult problem. In fact, the problem of finding good splits is not so different from the original problem of clustering the data, with the distinction that a split is a separation into only two, rather than many, clusters. Since we only need to divide the data into two clusters, we can take advantage of a common intuition that human operators have when performing splits: if a projection axis in the data exists which has a bimodal distribution, that is strong evidence that a split should be performed along that axis. This split can also be tested with respect to refractoriness of the CCG (like we check merges), and if the CCG is not refractory, then the split is typically performed.

How could we find such splits automatically? Human curators typically find the splits in a GUI like Phy, by investigating multiple scatter plots of pairs of principal components from a few neighboring channels. Clearly this can be improved on, since the optimal split should include information from all channels and all principal components. In Kilosort2, we designed an algorithm called bimodal pursuit to find projection axes that are highly bimodal. The "pursuit" part is a reference to other pursuit-type algorithms like ICA (kurtosis/skewness pursuit), and refers to the iterative nature of the algorithm which sequentially increases the bimodality of a projection $w^T \mathbf{x}$, where w is the unit-norm projection vector, and $\mathbf{x}$ is the data to be split into two clusters.

Specifically, we find the projection w which maximizes the following log-likelihood function:

$$
\begin{aligned}
\log \mathcal{L}(w) &= \sum_k \log p(x_k) \\
p(x_k) &= p_1 \mathcal{N}(w^t x_k; \mu_1, \sigma_1) + p_2 \mathcal{N}(w^t x_k; \mu_2, \sigma_2) \\
&= p_1 \frac{e^{-\frac{(w^t x_k - \mu_1)^2}{2\sigma_1^2}}}{\sqrt{2\pi\sigma_1^2}} + p_2 \frac{e^{-\frac{(w^t x_k - \mu_2)^2}{2\sigma_2^2}}}{\sqrt{2\pi\sigma_2^2}},
\end{aligned}
$$

where x_k are the features of the k-th spike, μ_j, σ_j are the scalar mean and variances of one cluster j out of the two total, p_j is the prior probability of drawing a spike from that cluster with $p_1 + p_2 = 1$. This

function can be optimized via an EM-style algorithm for mixtures of Gaussians, where we first infer the posterior distribution over cluster assignments, and then we optimize the free energy function with respect to p_j, μ_j, σ_j given w and viceversa. The posterior distribution over cluster assignments is given by the "responsibilities" $r_{kj} = \text{Prob}(y_k = j|\theta)$, where y_k is the true hidden label of data point k and θ is the set of all parameters.

$$r_{kj} = p_j \mathcal{N}(w^t x_k; \mu_j, \sigma_j)/p(x_k)$$

and the free energy function takes the form

$$\begin{aligned}
\mathcal{F}(\mathbf{r}, \theta) &= \sum_k \sum_j r_{kj} \log \mathcal{N}(w^t x_k; \mu_j, \sigma_j) \\
&= \sum_{k,j} r_{kj} \left(\log(p_j) - \frac{(w^T x_k - \mu_j)^2}{2\sigma_j^2} - \frac{1}{2}\pi\sigma_j^2 \right)
\end{aligned}$$

Holding w fixed, we can maximize with respect to p_j, μ_j, σ_j:

$$\begin{aligned}
p_j^{\text{new}} &= \sum_k r_{kj} / \sum_{k,j} r_{kj} \\
\mu_j^{\text{new}} &= \sum_k r_{k,j}(w^T x_k) / \sum_k r_{k,j} \\
(\sigma_j^{\text{new}})^2 &= \sum_k r_{k,j}(w^T x_k - \mu_j^{\text{new}})^2 / \sum_k r_{k,j}
\end{aligned}$$

Holding p_j, μ_j, σ_j fixed for all j, we can maximize with respect to w:

$$\begin{aligned}
w^{new} &= C^{-1} \left(\sum_k x_k \sum_j \frac{r_{kj}}{2\sigma_j^2} \mu_j \right) \\
C &= \sum_k x_k x_k^T \left(\sum_j \frac{r_{kj}}{2\sigma_j^2} \right)
\end{aligned}$$

w is re-normalized to unit norm on every iteration. The algorithm is initialized with w being either the top principal component of $\mathbf{x}$, or its normalized mean. In Kilosort 2 and 2.5, we run the algorithm twice, first initialized with the top principal component, and then initialized with the mean. The EM algorithm is run for 50 iterations, but w is only updated after iteration 10, and on odd iterations only, in order to make the optimization faster. We assign each spike k to the cluster y_k with highest posterior probability $r_{y_k k}$. We also compute a measure of the certainty in assigning y_k as: $q_j = < r_{jk} >_{y_k = j}$. If q_j is very close to 1, it means all spikes in cluster j are assigned with nearly maximum confidence. If it is close to its lower boundary of 0.5, it means there is almost no difference between the means and variances μ_j, σ_j of the two Gaussians in the mixture. To perform a split, we require that $\min(q_1, q_2) > 0.9$. In addition, we require that the resulting clusters have templates that are sufficiently distinct (correlation <0.9 or norms n_1, n_2 that are sufficiently different: $\|n_1 - n_2\|/(n_1 + n_2) > 0.1)$. We also require that the smallest cluster in the split should have at least 300 spikes, and that the cross-correlogram between resulting clusters is not refractory, using similar criteria to those described above for Kilosort4.

Splits are performed by traversing the list of clusters in consecutive order across channels. Once a split is found, we also check the subclusters for potential splits. We do this by appending the smallest sub-cluster to the end of the list, and testing the large cluster for splits again. This process continues until no more good splits are found, and then the process moves to the next cluster in the list.

Merges are also performed at the end in all versions of Kilosort starting with Kilosort2, and they take the some form as the global merges described above in the Kilosort4 section.

Recursive pursuit (Kilosort3)

In Kilosort3, we realized that the cost function above has some major weaknesses, such as the lack of scale invariance which means that projections with small amounts of variance have undesirably large values of $\log \mathcal{L}(w)$. Nonetheless, in practice the maximization of $\log \mathcal{L}(w)$ does indeed find projections with substantial bimodality, which is perhaps a consequence of good initialization and local minima. In Kilosort3, we made some appropriate modifications to the cost function as well as to the initialization to further improve its performance. The improved bimodal pursuit algorithm was able to find surprisingly good splits, even when given a mixture of more than two clusters. We took advantage of its performance and designed a new clustering algorithm in Kilosort3 which performs clustering by recursively splitting off clusters from the main distribution using the bimodal pursuit algorithm.

Improved bimodal pursuit

The idea of "projection pursuit" comes from the field of independent components analysis (ICA) and similar algorithms, and it was perhaps popularized the most by the fast ICA algorithm from Hyvarinnen and colleagues [1]. Like our cost function, projection pursuit maximizes some criterion computed over the distribution of projections $w^T x$. Unlike our function, this cri-

terion is usually scale-invariant, such as the kurtosis or skewness criteria which are normalized by the variance of the data. A simple way to make our criterion scale-invariant is whitening or "sphering", which is also a very common preprocessing step for ICA. Whitening normalizes a multi-dimensional dataset $\mathbf{x}$ into $\tilde{\mathbf{x}} = A\mathbf{x}$, where A is an appropriate whitening matrix, so that the mean of each dimension is 0, and the covariance of the normalized data is the identity. As a consequence, any projection $w^T\tilde{\mathbf{x}}$ is standardized, in other words it has mean 0 and variance 1. A common choice of whitening is PCA / SVD, and this is also our approach.

In Kilosort3, we perform whitening on every matrix $\mathbf{x}$ before running the bimodal pursuit algorithm. In this case, the log-likelihood criterion can be interpreted as searching for the projection w which can be best modelled by a mixture of Gaussians after z-scoring. Of all distributions with mean 0 and variance 1, the criterion $L(w)$ is now maximized by the sum of discrete distributions centered on -1 and $+1$. In addition, we constrain $\sigma_1 = \sigma_2 = \sigma$, which allows to perform the matrix inversion C^{-1} only once, because

$$\begin{aligned} C &= \frac{1}{\sigma}\sum_k x_k x_k^T \sum_j r_{kj} \\ &= \frac{1}{\sigma}\sum_k x_k x_k^T \\ &= \frac{1}{\sigma}N \end{aligned}$$

because $\sum_j r_{kj} = 1$ by construction and $\sum_k x_k x_k^T$ due to whitening, where N is the total number of spikes.

We also introduce a new form of initialization. Since the algorithm is highly sensitive to initialization, we run a brute force search for a good initialization vector w. Remembering that we are in normalized PCA space, the brute force approach checks all vectors w with $w_d \in \{-1/n, 1/n\}$ for $d = 1, ..., 6$ and $w_d = 0, d > 6$, with n being a normalization constant, in this case $\sqrt{6}$. For each of the resulting 64 combinations, we check the bimodality of the projection $w^T\mathbf{x}$ by performing a histogram and using similar criteria to those described above for Kilosort4. This histogram based algorithm is also used in the first 25 iterations of bimodal pursuit as a replacement for the EM assignments for μ, σ, p. This is done by computing the mean, variance and fraction of all points $w^T x_k$ smaller/bigger than the trough of the distribution respectively. We found this initial approximation of the EM assignments to be more robust, especially in cases where one cluster has substantially more spikes than the other.

Recursive pursuit

The bimodal pursuit algorithm described above takes as input a set of spike features, and outputs a partition into two clusters. Applied recursively, the algorithm can find a subset of a dataset that is well isolated from other clusters and cannot be split further into more clusters. We start will all spikes detected on a set of channels, and find the first split. Of the two pieces, we take the piece with higher average waveform norm, and we split it again. This process continues until no more splits can be found. Splits can be veto-ed in similar ways to Kilosort 2/2.5, except that the index of bimodality is used instead of the "measure of certainty" described above. A split requires all three criteria to be satisfied: high bimodality index, low waveform correlation and non-refractory CCG. In Kilosort4, we use the same criteria minus the criterion for low waveform correlation which is somewhat redundant with the bimodality index criterion.

Once a cluster is found out of the dataset, the spikes corresponding to that cluster are removed, and the cluster finding process is applied to the remaining spikes. This process continued until the remaining spikes can no longer be split, and thus they constitute the final cluster. Note that the complete algorithm contains two recursive loops: one loop for finding a single cluster out of the dataset, and another loop for finding all clusters in the dataset. This clustering operation is applied to spikes detected in 40 μm segments of the probe, similar to the process described for Kilosort4.

References

[1] Aapo Hyvarinen. Fast ica for noisy data using gaussian moments. In *1999 IEEE international symposium on circuits and systems (ISCAS)*, volume 5, pages 57–61. IEEE, 1999.
